# Antibiotic perturbation of gut bacteria does not significantly alter host responses to ocular disease in a songbird species

**DOI:** 10.7717/peerj.13559

**Published:** 2022-06-10

**Authors:** Chava L. Weitzman, Lisa K. Belden, Meghan May, Marissa M. Langager, Rami A. Dalloul, Dana M. Hawley

**Affiliations:** 1 Department of Biological Sciences, Virginia Polytechnic Institute and State University (Virginia Tech), Blacksburg, VA, United States of America; 2 Research Institute for the Environment and Livelihoods, Charles Darwin University, Darwin, Northern Territory, Australia; 3 Department of Biomedical Sciences, University of New England, Biddeford, ME, United States of America; 4 Department of Poultry Science, University of Georgia, Athens, GA, United States of America

**Keywords:** Disease ecology, Gut dysbiosis, House finch, *Mycoplasma gallisepticum*, Mycoplasmal conjunctivitis

## Abstract

Bacterial communities in and on wild hosts are increasingly appreciated for their importance in host health. Through both direct and indirect interactions, bacteria lining vertebrate gut mucosa provide hosts protection against infectious pathogens, sometimes even in distal body regions through immune regulation. In house finches (*Haemorhous mexicanus*), the bacterial pathogen *Mycoplasma gallisepticum* (MG) causes conjunctivitis, with ocular inflammation mediated by pro- and anti-inflammatory cytokines and infection triggering MG-specific antibodies. Here, we tested the role of gut bacteria in host responses to MG by using oral antibiotics to perturb bacteria in the gut of captive house finches prior to experimental inoculation with MG. We found no clear support for an impact of gut bacterial disruption on conjunctival pathology, MG load, or plasma antibody levels. However, there was a non-significant trend for birds with intact gut communities to have greater conjunctival pathology, suggesting a possible impact of gut bacteria on pro-inflammatory cytokine stimulation. Using 16S bacterial rRNA amplicon sequencing, we found dramatic differences in cloacal bacterial community composition between captive, wild-caught house finches in our experiment and free-living finches from the same population, with lower bacterial richness and core communities composed of fewer genera in captive finches. We hypothesize that captivity may have affected the strength of results in this experiment, necessitating further study with this consideration. The abundance of anthropogenic impacts on wildlife and their bacterial communities, alongside the emergence and spread of infectious diseases, highlights the importance of studies addressing the role of commensal bacteria in health and disease, and the consequences of gut bacterial shifts on wild hosts.

## Introduction

Symbiotic bacterial communities interact with invading pathogens in diverse ways and are increasingly recognized as important players in host health and disease (Daskin & Alford, 2012; Boon et al., 2014; Oliver, Smith & Russell, 2014; Becker et al., 2015). Beyond direct interactions influencing pathogen invasion and growth, commensal bacteria aid in host immune system development and regulate immune responses throughout life (Honda & Littman, 2012; Kabat, Srinivasan & Maloy, 2014; Lazar et al., 2018; Broom & Kogut, 2018; Kogut, Lee & Santin, 2020), indirectly affecting the strength and efficiency of hosts’ responses to pathogens. Bacteria along the mucosal surfaces of the gastro-intestinal tract (hereafter “gut”), in particular, provide hosts with diverse types of protection from infection, including serving as a physical barrier to pathogens on mucosal surfaces, and indirectly regulating host immunity in ways that often facilitate pathogen clearance (Khosravi & Mazmanian, 2013). On the other hand, gut bacteria can have complicated effects on host inflammatory immune responses, both in the gut and other tissues (Ichinohe et al., 2011; Hooper, Littman & Macpherson, 2012; Wilks & Golovkina, 2012; Shukla et al., 2017; Lazar et al., 2018; Kogut, Lee & Santin, 2020), making the effects of commensal gut bacteria on host responses to infections difficult to predict. As anthropogenic impacts on wildlife and their bacterial communities increase in extent, via antibiotics and other stressors (Kelly et al., 2014; Teyssier et al., 2018; Trevelline et al., 2019; Kraemer, Ramachandran & Perron, 2019; Lavrinienko et al., 2021), a better understanding of the importance of bacterial communities in wildlife hosts is imperative to prepare for, and predict, effects of emerging wildlife diseases.

While the importance of gut bacteria in health and disease has been established (Honda & Littman, 2012), the majority of studies focus on human health and mammalian model systems, with studies of wild and captive non-model species largely limited to characterizing gut communities within and among species (*e.g.*, Yuan et al., 2015; Cheng et al., 2015; Kohl et al., 2017; Teyssier et al., 2018; Song et al., 2020). Such studies broadly show that gut bacterial communities are influenced by both endogenous and exogenous factors and are maintained by interactions among hosts and between hosts and their environment (Harris, Roode & Gerardo, 2019; Koskella & Bergelson, 2020; Sarkar et al., 2020). For example, physiological stressors (Vlčková et al., 2018; Keohane et al., 2019), disease elsewhere in the body (Vieira & Pretorius, 2010; Wang et al., 2014), warming environmental temperatures (Bestion et al., 2017), and urbanization (Teyssier et al., 2018; Murray et al., 2020) can all influence gut microbial communities among individuals and populations. While the role of such gut microbial variation in wildlife health remains largely unknown (but see Davidson et al., 2021; Worsley et al., 2021), the growing number of factors shown to influence gut microbial composition in wild systems indicates that the consequences of such variation for wildlife health requires broader attention. These interactions are ideal for studying with controlled captive experiments to identify factors in natural systems that may require greater consideration in their effects on wildlife health. As such, captive studies of gut bacterial function in wild-caught animals can provide an important intermediate link between studies documenting gut microbial variation in free-living animals and experimental studies of gut bacteria and host health in lab animal models.

Few studies have directly examined the potential for gut bacteria in wild animals to influence host responses to pathogens, though studies of lab animal models suggest that such interactions are likely. Experiments in mice find that both innate and adaptive immune responses to respiratory viral infections are triggered by resident gut microbes (Ichinohe et al., 2011; Abt et al., 2012). Specifically, in response to experimental infection, mice with intact gut communities had shorter infection times, but increased inflammatory and antibody responses, compared with antibiotic-treated mice. In contrast, germ-free laboratory mice inoculated with microbiota from wild mice show reduced inflammation from influenza virus and increased survival (Rosshart et al., 2017). Similarly, mice given oral antibiotics to knock down gut microbes generally had greater inflammatory responses to *Streptococcus pneumoniae* infection than those with intact gut microbiomes (Schuijt et al., 2016). These results provide support for a key role of gut microbes mediating host inflammatory responses to pathogens. Importantly, there is evidence that diseases characterized by inflammatory responses may be particularly affected by gut bacteria, while gut microbes can be less important for non-inflammatory responses to infection. For example, pathogens that do not trigger inflammasome-dependent cytokine responses (*e.g.*, *Legionella pneumophila*) in hosts were not affected by knocking down the resident gut bacteria (Ichinohe et al., 2011). These experiments highlight the varied roles of gut communities in host disease, though a broader understanding of these interactions in non-mammalian and non-model systems is necessary.

In wild house finches (*Haemorhous mexicanus*), the ocular pathogen *Mycoplasma gallisepticum* (MG), which causes mycoplasmal conjunctivitis, has spread across much of the host’s distribution in the continental United States (Ley et al., 2016). Disease outbreaks in finches are associated with reduced host fitness and resulting population declines (Hochachka & Dhondt, 2000; Faustino et al., 2004). The degree of inflammation of the conjunctiva during infection is mediated by both pro- and anti-inflammatory cytokines (Vinkler et al., 2018). Because gut bacteria are mediators of immune responses in diverse vertebrates (Rosshart et al., 2017; Grond et al., 2018), they may be important during ocular infection in this system. Further, the conjunctival inflammation that house finches experience during infection appears to predict their likelihood of mortality from infection, which occurs largely via predation (Adelman, Mayer & Hawley, 2017); thus, understanding what factors drive variation in conjunctival inflammation, such as the gut bacterial community, is key for predicting the fitness impacts of this pathogen on host populations.

In this experiment, we tested the role of resident gut bacteria on disease in peripheral ocular tissues during mycoplasmal infection. We disrupted the gut bacteria through administration of oral antibiotics and then experimentally inoculated birds with MG to test the hypothesis that disrupted gut bacterial communities would affect disease caused by MG. We predicted that if gut bacteria stimulate pro-inflammatory cytokine production in this system, then antibiotic-treated birds would show less severe conjunctival inflammation, given documented associations between conjunctivitis severity and pro-inflammatory cytokine expression (Vinkler et al., 2018). Alongside reduced pathology, we expected to find reduced MG-specific antibody production, higher mycoplasmal loads, and longer disease, as detected in other systems (Abt et al., 2012). Alternatively, because pathology is coincident with mycoplasmal tissue damage, we considered that we could find increased pathology in antibiotics-treated birds. To address the hypothesis that we could detect shifts in gut bacteria due to antibiotics administration, we used amplicon sequencing of cloacal swabs as a proxy for mucosal bacteria in the gut to avoid destructive sampling of hosts that we were actively monitoring for conjunctival disease outcomes. Lastly, to put our captive experiment on this wild-caught species into context, we compared cloacal swabs from our experimental birds to free-living birds to assess the differences in bacterial communities in captive versus wild house finches.

## Materials & Methods

### Bird capture and housing

**Figure 1:**
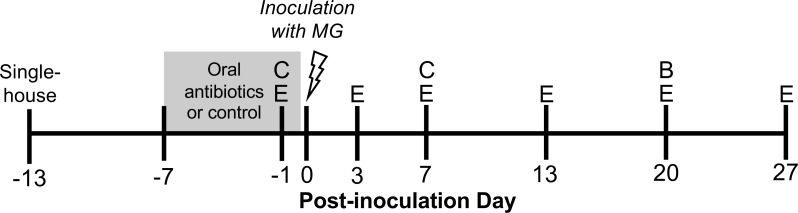
Experimental timeline. Wild-caught house finches were administered oral antibiotics for one week prior to inoculation with *Mycoplasma gallisepticum*. Throughout the course of disease, we monitored pathology and pathogen load by scoring eye lesions and collecting eye swabs (E) and detected differences in antibody responses with blood samples (B). We also collected cloacal swabs to detect short- and long-term effects of oral antibiotics on cloacal bacterial communities (C). A subset of cloacal swabs were 16S rRNA gene amplicon sequenced (see Table 1).

Hatch-year house finches (*n* = 50) were wild-caught in June–July 2020 in Blacksburg, Montgomery County, Virginia. Upon capture, finches were housed singly or in pairs and subjected to a two week quarantine period, during which time we verified that no bird exhibited clinical signs of conjunctivitis. After two weeks, we also tested for MG-specific antibodies with enzyme-linked immunosorbent assay (ELISA; Hawley et al., 2011), and no seropositive birds were included in the present study. To minimize mortality due to coccidiosis, all birds were treated with Endocox (toltrazuril) in their water (1.29 g/L for three days every one to four weeks). Endocox treatment should not have directly affected the gut bacteria (Maurice et al., 2015), and treatment ceased 18 days before MG inoculation. Birds were single-housed 13 days prior to inoculation, at the end of September 2020 (Fig. 1), and were provided a constant 12:12 light-dark cycle and food and water *ad libitum.* Birds were captured under VDGIF (066646) and USFWS (MB158404-0) permits. Experimental procedures were approved by Virginia Tech’s Institutional Animal Care and Use Committee (BIOL-18-144).

### Experimental design

Experimental birds were randomly assigned to oral antibiotics and MG treatments in a 2 × 2 factorial design, with as close to 50:50 sex ratio as possible per treatment group. To perturb the gut bacteria with antibiotics, we administered a mixture of amoxicillin and metronidazole in the birds’ water (available *ad libitum*), at final concentrations of 1,000 mg/L and 200 mg/L respectively, for seven days prior to MG inoculation (Dorrestein, 2009, *n* = 25 birds, Fig. 1, Table 1). Water (control) and antibiotics were refreshed daily. The length of these broad-spectrum antibiotics administration was chosen based on studies in chickens to cause large enough changes to affect immune responses (Wise & Siragusa, 2007; Pélissier et al., 2010; Pallav et al., 2014; Schokker et al., 2017; Connelly et al., 2018; Xiong et al., 2018). Though these antibiotics may be spread systemically through the bird, they both have short half-lives in other birds (≤12 h in poultry; Anadón et al., 1996; Cybulski et al., 1996), and we ceased antibiotics treatment prior to MG inoculation. Further, any residual antibiotics present in birds would not affect MG itself as they mainly affect bacteria with cell walls (amoxicillin) and anaerobic bacteria (metronidazole) (Anadón et al., 1996; Bébéar, Pereyre & Peuchant, 2011; Ceruelos et al., 2019). On MG inoculation day, we administered 70 µL of Frey’s media (control, Table 1) or MG diluted in Frey’s media (VA1994 isolate, 7994-1 6 P 9/17/2018) to a concentration of 3 × 10^4^ color changing units/mL (CCU/mL) by droplet instillation onto the conjunctivae.

**Table 1:** Experimental design. House finches were randomly assigned to receive oral antibiotics in their water and inoculated with *Mycoplasma gallisepticum* (MG) or sham control. Values in parentheses represent sample sizes for 16S rRNA gene amplicon sequencing of cloacal swabs. These cloacal swab samples were collected on day –1 (after oral antibiotics but prior to MG inoculation), with MG sham control samples from day 7 also sequenced to detect the extended effects of oral antibiotics.

**Sample sizes:**	**MG control (sham)**	**MG**
Microbiome control (no antibiotics)	*n* = 8 (8)	*n* = 17 (12)
Antibiotics	*n* = 8 (8)	*n* = 17 (5)

We focused our data collection on following disease in birds throughout infection, making us unable to measure cytokine expression in this study, which requires destructive sampling (Vinkler et al., 2018). Birds were monitored for pathology and MG load periodically throughout infection (Fig. 1). We scored the degree of pathology for each conjunctiva on a scale of 0–3 and summed the values between the two eyes for a given sampling day (Sydenstricker et al., 2005). No experimental birds had any pathology prior to MG inoculation. To measure MG loads, cotton-tipped swabs were lubricated with tryptose phosphate broth (TPB) before gently swabbing each conjunctiva and wringing out the swab in a collection tube of 300 µL TPB. Both conjunctival swab samples per bird were combined into a single TPB tube. Both eye scoring and swabbing were done by a single individual (CLW), blind to treatment.

### Measuring pathogen load

We extracted DNA from a subset of ocular swab samples from MG-treated birds on post-inoculation days –1, 3, 7, and 13 (*n* = 8–10 per treatment per day; Table S1) using the Qiagen DNeasy 96 Blood and Tissue Kit (Qiagen, Valencia, CA) to examine differences between the treatment groups in MG loads early in infection. We also extracted samples from sham MG birds on day 3 to ensure that our sham controls were MG-free, because day –1 samples from these birds were used for a separate study. We included four extraction controls (extraction reagents with no sample) on the DNA extraction plate interspersed among our samples. MG load was measured with a probe-based quantitative polymerase chain reaction (qPCR) amplifying the MGC2 gene, as previously described (Grodio et al., 2008; Leon & Hawley, 2017). With every MGC2 qPCR plate run we included a standard curve of eight plasmid concentrations (10^1^ to 10^8^ copies) in triplicate.

**Figure 2:**
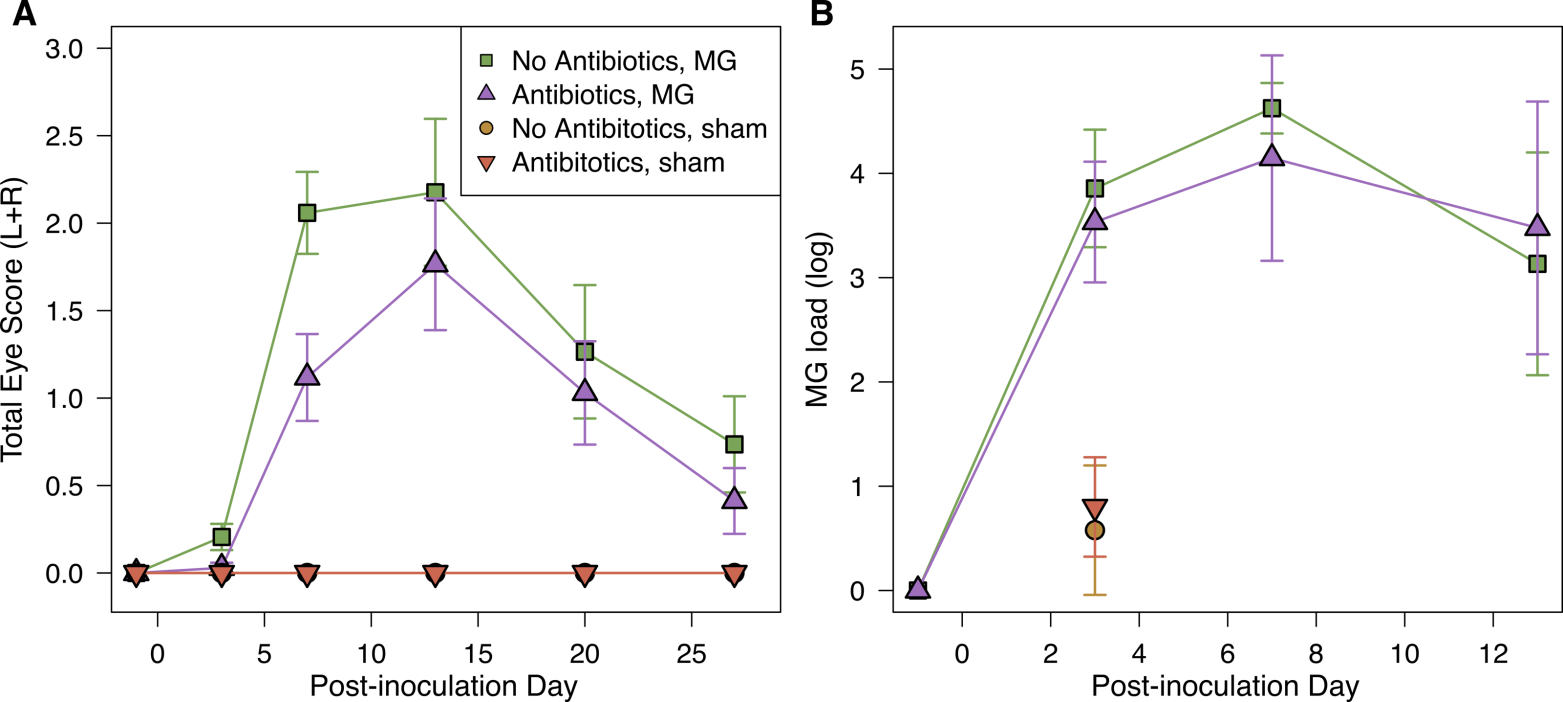
Mycoplasmal disease in house finches was not significantly affected by pre-inoculation oral antibiotics. (A) Pathology eye scores (left plus right eye scores) and (B) MG loads (log_10_(load+1)) in the conjuctiva by experimental treatment group (*n* = 8–10 per point, Table S1) across the course of disease. MG loads for sham-inoculated birds were only measured on day 3 post-inoculation. Values are averages ± standard error of raw data.

We verified with ocular swab samples from day –1 that birds in MG inoculated groups did not begin the experiment with active MG infections; those samples were negative for MG via qPCR. Some of the sham control bird samples from day 3 were positive for low levels of MG via qPCR (max of 10^1.66^ copies; Fig. 2B), but the lowest value from a MG-inoculated bird on that post-infection day (10^2.55^ copies) was 8-times greater than the highest MG sham control sample. These positive MG sham control samples were likely contaminated during DNA extraction, supported by the absence of pathology for any MG-control birds throughout the experiment. The qPCR reaction we use is very sensitive to low levels of contamination, which have been detected previously in this system (Leon & Hawley, 2017). Because treatment groups were randomized among the extraction plate, low-level contamination is not likely to affect our results. None of the extraction controls tested positive for MG from qPCR.

### Bacterial community characterization

In birds and many other vertebrates, the cloaca is the terminal opening for the digestive, urinary, and reproductive tracts, making cloacal swabbing a minimally invasive method standardly used in avian studies for collecting microbial communities (Klomp et al., 2008; Escallón et al., 2017). We used amplicon sequencing of cloacal swab samples to address the hypothesis that we could detect shifts in the gut bacterial communities due to antibiotics with cloacal swab data, as well as the hypothesis that cloacal bacteria in captive house finches are representative of those in wild house finches.

On day –1 post-inoculation (day 6 of antibiotics treatment) and day 7 post-inoculation (Fig. 1), we gently inserted a sterile swab (PurFlock Ultra^®^, Puritan, Guilford, Maine) ∼4mm into the cloaca and rotated for 5 s. Swabs were placed in sterilized 1.5 mL microcentrifuge tubes on ice after collection and frozen at −80 ° C until DNA extraction. We extracted DNA from 49 cloacal swab samples (Table 1) with the Qiagen DNeasy Blood and Tissue Kit protocol for Gram-positive bacteria. We focused DNA extraction on detecting effects of antibiotics and avoiding confounding effects of MG infection, extracting both sample dates for all 16 MG control birds and only day –1 samples from birds subsequently given MG. To compare cloacal bacterial communities from captive birds with those in the wild from the same population, we also extracted DNA (using identical methods) from 16 cloacal swab samples collected from wild house finches in October–November 2020 in Blacksburg, VA.

Unlike the extraction methods detailed above for ocular swab samples (96-well extraction), our DNA extractions of cloacal samples were conducted in a biosafety cabinet with single-tube extractions to minimize contamination into and among the samples. Alongside the cloacal swab extractions, we extracted DNA from environmental controls (*n* = 4; exposing a swab to the air) and extraction controls (*n* = 5), and conducted library prep on *n* = 6 of these control samples, but did not include them in the sequence run. From Qubit analysis, average ± SD ng/µL of library prepped cloacal samples and controls were 16.7 ± 10.4 and 2.2 ± 1.0 respectively. Thus, we are confident that the bacterial communities described here largely constitute those in the cloaca and not from outside sources.

For cloacal swab bacterial community assessment, we amplified the V4 region of bacterial 16S rRNA gene with the 515F/806R primers (Caporaso et al., 2012) as previously described (Thomason et al., 2017; Hernandez et al., 2020; Weitzman et al., 2021), and amplicons were sequenced using a 250 bp single-end strategy on an Illumina MiSeq at the Dana Farber Cancer Center of Harvard University. Forward reads were demultiplexed in QIIME2 (Bolyen et al., 2019) and processed with the DADA2 package with R version 4.0.2 in RStudio version 1.3.1093 (R Development Core Team, 2015; Callahan et al., 2016; RStudioTeam, 2020)). With the filterAndTrim function, we kept reads with a minimum length of 250 bases and 5 maximum expected errors. After inferring amplicon sequence variants (ASVs), we removed chimeras, assigned taxonomy with the Silva v132 database using the assignTaxonomy command, and removed non-bacterial, mitochondrial, and chloroplast reads. After inspection of rarefaction curves, we rarefied samples to 5,000 reads for richness and alpha diversity analyses and kept unrarefied data from samples with a minimum of 1,000 reads for beta diversity analyses and analysis of differential abundance. Beta diversity metrics were calculated from data transformed to proportional reads (on a per sample basis, reads per ASV divided by total reads; McKnight et al., 2019). We used QIIME2 to determine ASV richness, Faith’s phylogenetic diversity, Bray–Curtis and Jaccard distances, and weighted and unweighted UniFrac distances. UniFrac reflects phylogenetic-based distances, with unweighted UniFrac distances based off presence-absence data and weighted UniFrac distances incorporating relative abundance. From these diversity metrics, we calculated the change in richness and phylogenetic diversity per bird where applicable (day 7 post-inoculation –day –1 pre-inoculation) and also extracted pairwise distance values of samples between day –1 and day 7 post-inoculation per bird. For analyses of differential abundance, we collapsed the ASV table to genus level.

### Statistical analyses

Statistical analyses were run with R version 4.0.2 in RStudio version 1.3.1093 (R Development Core Team, 2015; R Studio Team, 2020). The full models for pathology, MG load, and probability of infection included antibiotics treatment, post-inoculation day, their interaction, sex, and a random variable of bird ID. We used model simplification to arrive at final models, removing the interaction term or covariates (day, sex) when *p* > 0.1 from Wald’s tests in the car package (Fox & Weisberg, 2019). We used linear mixed-effects models (LMM) with the lme4 package (Bates et al., 2015), analyzing pathology data from MG-inoculated birds only, to test the hypothesis that oral antibiotics influenced the degree of house finch conjunctival pathology after inoculation with MG. To fit assumptions of normality in analyses, we analyzed pathology data as log(sum eye score + 1). We similarly analyzed MG load, as log_10_(load + 1), with LMM to test the hypothesis that oral antibiotics treatment influenced MG growth. Here we modeled post-inoculation day as an ordinal variable because MG load was only measured on three post-inoculation days. We additionally analyzed whether the probability of infection (Y/N) differed based on antibiotics treatment, defining successful infection as a conjunctival MG load >10^3.1^ copies (Adelman et al., 2015; Leon & Hawley, 2017). Because a subset of samples were randomly extracted for each post-inoculation day, we qualified each MG load as “infected” or not and analyzed probability of infection with a binomial generalized linear model with a probit link, with day as an ordinal variable as above.

We used 16S rRNA amplicon sequences from cloacal swabs to address two additional hypotheses: oral antibiotics affect cloacal bacterial communities, but communities begin to return to their initial composition one week after antibiotics treatment ends; and cloacal bacteria in captive house finches are similar to, and representative of, those in wild house finches.

To assess the effects of antibiotics on cloacal bacterial communities, we subset the alpha and beta diversity metrics, and their changes over time, to only include captive birds in the experiment. We made three sets of comparisons with these data: differences between communities on day –1 in birds that were given antibiotics and non-treated birds; changes in communities within birds from day –1 to day 7; and differences between communities on day 7 in antibiotics and non-treated control birds. We analyzed log-transformed values of ASV richness and Faith’s phylogenetic diversity with LMM, determining *p*-values with Wald’s tests. Analyses included predictor variables of antibiotics treatment, post-inoculation day (categorical with two sampling days), their interaction, sex, and the random variable of bird ID. We assessed differences in bacterial community structure based on the same predictor variables with permutational analysis of variance (PERMANOVA) with a block design (bird ID as random variable) and analysis of beta dispersion in the vegan package (Oksanen et al., 2020), and visualized community structure differences with principal coordinates analysis in the ape package (PCoA; Paradis & Schliep, 2019). ANOVAs were used to compare changes in richness and phylogenetic diversity, and pairwise beta diversity distances (distance value within a bird between day –1 and day 7), between antibiotics treatment groups. Though we initially included sex as a covariate in each of these analyses, it was never significant and was removed for final results.

We then used genus-level data to detect differentially abundant taxa between antibiotics treatments and sampling days with ALDEx2 (Fernandes et al., 2013; Fernandes et al., 2014) in QIIME2, with genera present in at least 10% of the samples included in each analysis. Genera were considered differentially abundant when Wilcoxon rank test *p*-value was below 0.05 with a Benjamini–Hochberg correction (Benjamini & Hochberg, 1995). ALDEx2 can only perform pairwise comparisons between two groups, so we subset and analyzed our data with this consideration, comparing relative abundances of genera between: antibiotics and non-treated birds on day –1 (antibiotics *n* = 12, control *n* = 19), day –1 (*n* = 12) and day 7 (*n* = 8) in birds given antibiotics, and birds given antibiotics compared with non-treated birds on day 7 (*n* = 8 each).

To detect whether cloacal communities of captive finches are representative of those of wild birds, we subset the cloacal swab data to include samples from non-antibiotics-treated birds from the experiment on day –1 and samples from wild birds. Thus, no birds in this had received oral antibiotics. We compared log-transformed richness and phylogenetic diversity between these groups (captive versus wild finches) with ANOVAs. We also used PERMANOVA and analysis of beta dispersion to compare beta distances, as well as ALDEx2 to detect differentially abundant genera between these two groups of samples. Finally, we determined the overlap of core genera (present in >85% of samples per group) between these groups.

## Results

### Infection and disease

Inoculation with MG resulted in varied host responses, with all but three inoculated birds exhibiting some visible conjunctival pathology during the experiment. The severity of pathology did not differ based on pre-inoculation oral antibiotics treatment (LMM, estimate ± SD = −0.19 ± 0.12, *χ*^2^ = 2.35, *p* = 0.1); covariates of post-inoculation day (and its interaction with antibiotics treatment) and sex were removed from the final model because they were not significant. Although not significant, average differences in pathology followed our predictions, with a lower average degree of conjunctival inflammation in hosts given antibiotics treatment prior to infection compared to controls, particularly early in infection (Fig. 2A).

MG load differed by post-inoculation day, but not by antibiotics treatment (LMM, day: *χ*^2^ = 24.40, *p* < 0.0001; antibiotics treatment: estimate ± SD = −0.14 ± 0.26, *χ*^2^ = 0.27, *p* > 0.5; Fig. 2B). We also assessed the probability of being infected (binomial Y/N) on the post-inoculation days for which we had MG load data, and similarly found that the probability of being infected differed significantly among post-inoculation days, but not based on antibiotics treatment (day: *χ*^2^ = 11.75, *p* = 0.003; antibiotics treatment: estimate ± SD = 0.08 ± 0.39, *χ*^2^ = 0.04, *p* > 0.5). Finally, we did not find any significant effects of treatment on plasma antibody levels in response to experimental infection (Supplemental Information).

### Cloacal bacteria in captive house finches

**Figure 3:**
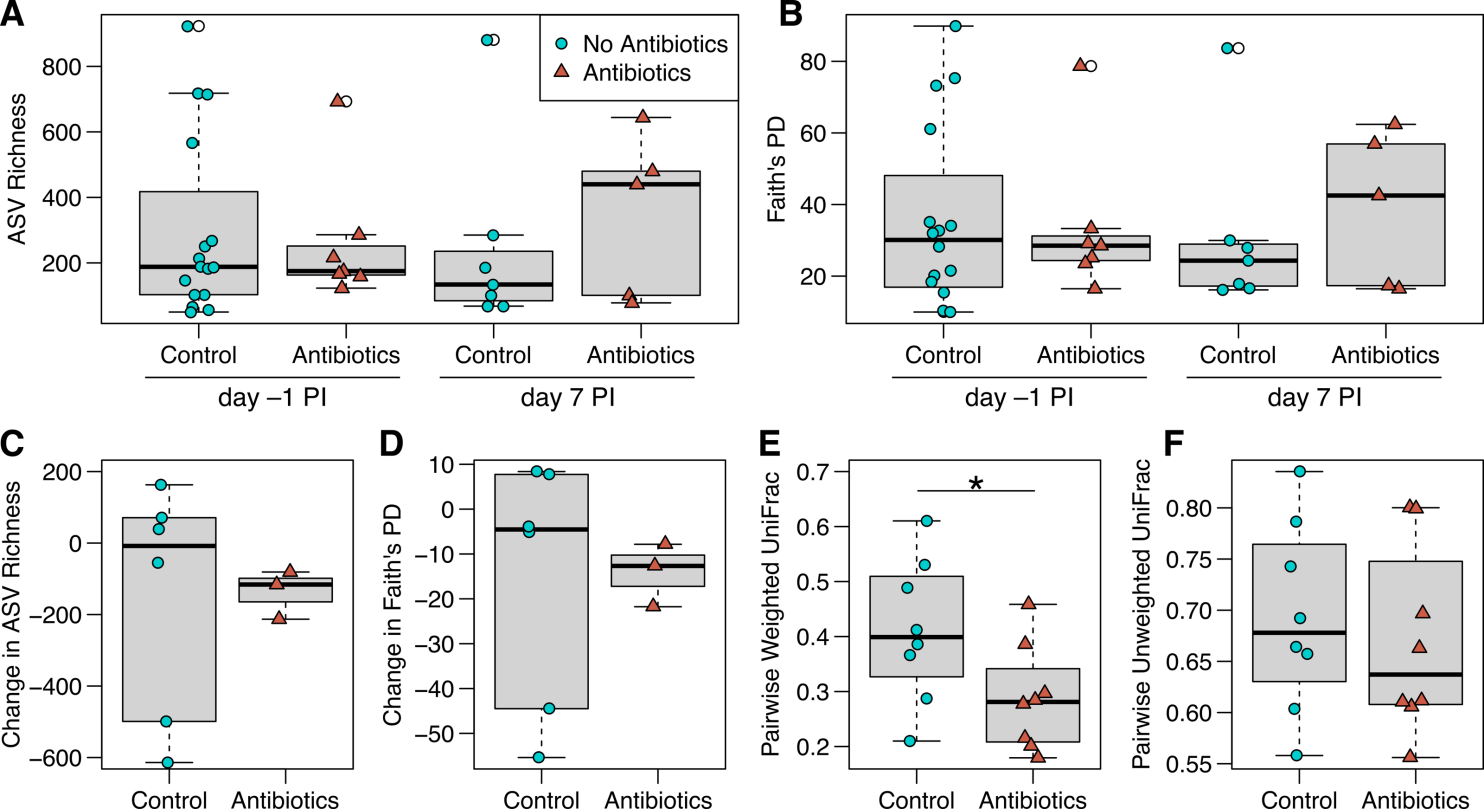
ASV richness and phylogenetic diversity in captive house finch cloacal swab samples. (A) Richness and (B) Faith’s phylogenetic diversity by antibiotics treatment group and day sampled, with day –1 post-inoculation (PI) representing the sixth day of antibiotics or control treatment (day –1 control *n* = 16, antibiotics *n* = 7; day 7 control *n* = 7, antibiotics *n* = 5). (C, D) Change in richness and phylogenetic diversity from day –1 to day 7 post-inoculation within each individual bird (control *n* = 6, antibiotics *n* = 3). (E, F) Pairwise weighted and unweighted UniFrac distances within individuals from day –1 to 7 (*n* = 8 per group), where greater values indicate less similarity. Boxplots indicate the median, interquartile range, reasonable range of the data, and outliers (open circles).

In the total 65 cloacal swab samples (including samples from 16 wild birds), we detected 9,460 bacterial ASVs from 1,951,109 total bacterial reads (102–116,421 reads per sample, mean = 30,019 ± 31,073). We removed two samples with fewer than 1,000 reads (one sample from each: day –1 MG treatment, day –1 both antibiotics treatment+MG). Rarefaction removed an additional 12 samples from alpha diversity analyses (Fig. 3). The cloacal communities in our captive house finches at the beginning of the experiment were largely comprised of Proteobacteria (*e.g.*, *Pseudomonas*, *Sphingomonas*, *Janthinobacterium*) and Firmicutes (*e.g.*, *Candidatus Arthromitus*, *Staphylococcus*, *Bacillus*), followed by Actinobacteria, and Tenericutes (Fig. S1).

We did not detect strong, immediate effects of antibiotics on cloacal bacterial communities. At day –1 (the final day of oral antibiotics), diversity metrics were largely comparable for non-treated and antibiotics-treated birds (Figs. 3 and 3B). However, antibiotics-treated birds appeared to have less variable bacterial diversity than did non-treated birds at day –1, just prior to MG inoculation, though this pattern reversed on day 7 post-inoculation (Fig. 3 and 3B). Overall, there was no significant effect of antibiotics treatment on bacterial richness (estimate ± SD = 0.26 ± 0.32, *χ*^2^ = 0.68, *p* = 0.4 ; Fig. 3A), phylogenetic diversity (estimate ± SD = 0.18 ± 0.24, *χ*^2^ = 0.56, *p* = 0.5 ; Fig. 3B), unweighted UniFrac distance (sum of squares = 0.25, *F*_1,45_ = 0.98, *R* ^2^ = 2.1%, *p* > 0.5; Fig. S2) or weighted UniFrac distance (sum of squares = 0.29, *F*_1,45_ = 2.12, *R* ^2^ = 4.5%, *p* > 0.5, Fig. S2). The difference in community structure based on antibiotics treatment was not explained by differing dispersion (unweighted UniFrac: sum of squares = 0.003, *F*_1,45_ = 1.27, *p* = 0.3; weighted UniFrac: sum of squares = 0.03, *F*_1,45_ = 1.94, *p* = 0.2). Beta diversity based on Bray–Curtis and Jaccard distances were similarly not affected by antibiotics treatment (Table S2, Fig. S2). Post-inoculation day, the interaction between day and antibiotics treatment, and host sex were not significant in any comparisons of bacterial communities and were removed from all analyses.

When examining within-individual changes in diversity metrics from day –1 to day 7 post-inoculation, antibiotics treatment significantly predicted paired weighted UniFrac distances (sum of squares = 0.06, *F*_1,14_ = 4.73, *p* = 0.047; Fig. 3E), with results indicating that samples from birds given antibiotics were more similar between the sampling days than control samples from birds that were not treated. However, we found no effect of antibiotics treatment on within-individual changes over time with respect to most other diversity metrics (Fig. 3; richness: *F*_1,7_ = 0.004, *p* = 1; phylogenetic diversity: *F*_1,7_ = 0.007, *p* = 0.9; paired unweighted UniFrac: *F*_1,14_ = 0.29, *p* = 0.6; paired Bray-Curtis and Jaccard results in Table S2). Antibiotics-treated birds showed a nonsignificant decline in richness and phylogenetic diversity over time, which could represent a delayed effect of antibiotics, though samples from birds not given antibiotics were highly variable.

We used ALDEx2 to detect differentially abundant genera in cloacal bacterial communities between antibiotics treatments and sampling days, but found no differences between the birds given antibiotics and non-treated control birds on day –1 or day 7, and no differences in antibiotics-treated birds between day –1 and day 7.

### Captive *vs* wild house finch bacteria

Cloacal samples from captive birds (in the lab 10+ weeks prior to the experiment) that did not receive oral antibiotics had significantly different cloacal bacterial communities compared with birds sampled in the wild at the same time of year (Table S3). Importantly, wild samples had higher ASV richness, and the communities differed in community structure (Table S3, Figs. S3, S4). In ADLEx2 analyses, we found 76 genera (out of 621, 12.2%) to be differentially abundant in cloacal communities between untreated captive birds (day –1) and wild samples (Table S4), most of which (*n* = 62) were more abundant in wild bird samples. We further found that samples from birds in the lab had simpler core bacterial communities, with fewer prevalent genera than cloacal samples from wild birds (Fig. S5).

## Discussion

Experimental and observational studies are increasingly finding that infectious disease outcomes in hosts are affected by resident bacterial communities (Hooper, Littman & Macpherson, 2012; Weyrich et al., 2014; Walke & Belden, 2016; Rosshart et al., 2017). We used oral antibiotics and experimental MG inoculation to quantify indirect effects of gut bacteria on ocular disease in house finches. Though we did not find treatment of the gut bacterial microbiome with antibiotics to significantly affect MG pathology, pathogen load, or antibody responses, antibiotics-treated birds had slightly lower average pathology than did control birds early in infection, though it was not a great enough difference to significantly affect the treatment-by-day interaction in analyses. Further, we detected stark differences in cloacal bacterial communities between captive house finches and those in the wild. We focus our discussion of these experimental results through the lens of bacterial communities in our birds.

### Gut bacteria and ocular disease

As in other systems (Bornbusch et al., 2021), we expected to find long-term effects of gut bacterial disruption on immune function and consequently disease response. In this experiment, oral antibiotics prior to infection did not strongly affect ocular pathology after inoculation with a high dose of MG, though the data show a slight trend of antibiotics treatment reducing the average degree of pathology early in infection. Though not significant, an average reduction in early ocular inflammation could indicate that some gut bacteria affected by antibiotics treatment in our study may stimulate the inflammatory immune response. House finches experience an influx of local and systemic pro-inflammatory cytokine production (IL-1β, IFN-γ, TNF-α) early in infection with MG (Adelman et al., 2013; Vinkler et al., 2018), and individual variation in conjunctivitis is significantly predicted by individual variation in local IL-1β expression. In this experiment, we were unable to measure cytokine expression, which requires destructive sampling and thus precludes the ability to track infection outcomes. However, an experimental study in mice found that antibiotics to knock down gut bacteria resulted in decreased inflammatory cytokine production and decreased clinical signs of experimental autoimmune uveitis, a non-infectious ocular disease (Nakamura et al., 2016). Similarly, the gut microbiota in mice has been shown to induce inflammatory immune responses to respiratory infection (Ichinohe et al., 2011), indicating that gut microbiota can stimulate inflammatory responses at diverse mucosal surfaces.

In our birds, the reduced average inflammation after gut bacterial perturbation was negligible (∼0.5 pathology score reduction), which, even if statistically significant, would not likely result in dramatic differences in the bird’s vision and ability to find food or evade predators. However, our characterization of cloacal communities indicates that the bacteria in captive house finches may not have been representative of those in wild birds, having lost many bacteria that were prevalent in wild birds. Consequently, gut bacteria may have greater importance for disease outcomes in wild birds than their captive counterparts. Alternatively, gut bacteria in house finches may not play an important role in inflammatory responses in peripheral tissues. However, the close association between gut bacteria and the bursa of Fabricius, the site of B cell development in birds located within the intestinal tract, suggests that intestinal bacteria should have immunomodulatory effects, particularly in the context of cytokine-mediated inflammation (reviewed in Kohl, 2012). As a nascent field, we cannot compare our results with others from songbirds, though the field is gaining traction in human and mouse models, particularly regarding nonpathogenic ocular diseases. For example, in addition to the mouse experiment noted above (Nakamura et al., 2016), an observational study in people with non-pathogenic uveitis found shifts in gut communities, including decreased diversity, compared with healthy subjects (Kalyana Chakravarthy et al., 2018). Overall, while we did not find statistical support for effects of gut bacteria in altering host responses to ocular disease in house finches here, studies of local and systemic cytokine responses may still be warranted, particularly early in infection when differences in inflammatory immune responses appear important in driving host disease outcomes (Adelman et al., 2013).

### Describing and disturbing gut bacteria

The absence of detectable effects of gut bacteria on host responses in our study may have at least partly resulted from limitations in our ability to meaningfully perturb the gut microbiome in captive house finches. Through amplicon sequencing, we detected dramatic differences between the cloacal bacteria of our captive house finches and those sampled in the wild at the time of our study. Notably, wild finches had greater cloacal bacterial richness, with greater relative abundance of many genera when compared with captive finches (Fig. S3). Captivity can affect host-associated bacterial communities due to changes in environment, diet, and stressors (Bailey et al., 2010; Becker et al., 2014; Clayton et al., 2016; Bates et al., 2019), and captivity was found to affect bacterial communities in other birds (*e.g.*, Oliveira et al., 2020; San Juan, Castro & Dhami, 2021). However, one study suggests that birds in particular tend to have large quantities of transient bacteria (Song et al., 2020), which may account for a portion of the differences we found between our captive and wild samples. Conducting this experiment on wild-caught birds ensured that their starting communities at least partially represented those of wild birds; however, months in captivity may have affected the bacteria enough to affect our ability to fully test their importance in mycoplasmal conjunctivitis in wild house finches.

In the limited laboratory environment, it is not surprising that microbial diversity was significantly lower when compared to birds in the wild. Some captive birds appeared to lose hundreds of bacterial ASVs between the sampling days, possibly because the communities were still shifting as a result of being single-housed. In another study, captive ptarmigans not only had different cecal bacterial community composition, but also a lower total number of bacterial cells per cecal weight when compared with wild ptarmigans (Salgado-Flores et al., 2019). In laboratory mice, microbiome-mediated immune protection was restored when germ-free mice were recolonized with wild, but not lab mouse gut communities (Rosshart et al., 2017). Interestingly, a large comparative study found that birds tend to have lower bacterial counts in their feces, suggesting overall lower resident bacterial microbiome biomass (Song et al., 2020). For birds with low gut bacterial biomass, it is possible that few taxa provide the majority of the benefit of gut communities. Future studies on house finches with more natural gut communities may better reveal effects of gut microbes on host responses.

The changes in gut bacteria due to captivity may not have just affected its protective role; they may have also hampered our ability to use antibiotics to thoroughly test the bacteria’s role in protection, if captive birds harbor a less abundant and less complex microbiome. From amplicon sequencing of cloacal bacteria, we detected subtle but not strong differences in community structure or composition based on antibiotics treatment. Interestingly, we found that birds given antibiotics had more similar communities over an eight-day period (day –1 to day 7) than those that did not receive antibiotics, based on weighted UniFrac distances. While data from cloacal samples as a proxy for gut bacteria should be interpreted with caution, this suggests that changes in cloacal bacteria due to the antibiotics prior to inoculation may have lasted well beyond inoculation day. Strikingly, however, we found no indication of reduced cloacal bacterial diversity in antibiotic-treated relative to control birds, suggesting that oral antibiotics may not have had strong effects on gut bacterial diversity in captive house finches. Another study in chickens, which used antibiotic administration to investigate the importance of gut bacteria on immune development, also only found minimal detectable changes in amplicon sequencing of luminal bacteria (Schokker et al., 2017). On the other hand, our ability to detect differences in bacterial diversity was limited by our sampling procedures, which were more likely to detect luminal bacteria than those with a close association with gut mucosal surfaces. Thus, we may have missed important effects of antibiotics on mucosal bacteria, which were found to be more affected by oral antibiotics than luminal bacteria in another songbird system (Kohl et al., 2018). Further, cloacal swabbing picks up bacteria leaving the gut, but is not always a reliable method of measuring bacteria higher in the intestinal tract, including the colon, cecum, and ileum (Videvall et al., 2018). However, preliminary data from cloacal and colon swab samples from captive house finches show that cloacal swabs do loosely represent communities in the lower gastro-intestinal tract (see Supplemental Information). Further study using destructive sampling should examine effects of antibiotics on gut communities directly, which may shed more light on any changes in gut microbes that resulted from antibiotics treatment and their potential role in host responses to disease.

## Conclusions

While we did not find strong evidence for effects of gut bacteria on mycoplasmal conjunctivitis in captive house finches, the nonsignificant trend of decreased pathology after antibiotics indicates that further experiments are warranted. Experiments with house finches sooner after capture, and with the addition of cytokine expression data collection from several tissues including the gastro-intestinal tract, may more definitively reveal whether and how gut bacteria play a role in the individual- and population-level effects of MG found in wild populations.

## Supplemental Information

10.7717/peerj.13559/supp-1 Supplemental Information 1 Supplemental Materials: Additional methods and results for the main experiment (Table S1–S4 and Figs. S1–5)

10.7717/peerj.13559/supp-2 Supplemental Information 2 Supplemental Materials: Methods and results of cloacal and intestinal swab sampling and sequencing from house finches conducted in 2014/2015 (Table S5 and Fig. S6)

10.7717/peerj.13559/supp-3 Supplemental Information 3 Pathology, pathogen load, and antibody data from house finches inoculated with *Mycoplasma gallisepticum* Raw data and column descriptions

## Additional Information and Declarations

### Field Study Permissions

The following information was supplied relating to field study approvals (i.e., approving body and any reference numbers): Birds were captured under VDGIF (066646) and USFWS (MB158404-0) permits.

### DNA Deposition

The following information was supplied regarding the deposition of DNA sequences: The amplicon sequences are available in the NCBI Sequence Read Archive: PRJNA773380.

### Data Availability

The following information was supplied regarding data availability: The raw data are available in the Supplementary Files.

## References

[ref-1] Abt MC, Osborne LC, Monticelli LA, Doering TA, Alenghat T, Sonnenberg GF, Paley MA, Antenus M, Williams KL, Erikson J (2012). Commensal bacteria calibrate the activation threshold of innate antiviral immunity. Immunity.

[ref-2] Adelman JS, Kirkpatrick L, Grodio JL, Hawley DM (2013). House finch populations differ in early inflammatory signaling and pathogen tolerance at the peak of *Mycoplasma gallisepticum* infection. The American Naturalist.

[ref-3] Adelman JS, Mayer C, Hawley DM (2017). Infection reduces anti-predator behaviors in house finches. Journal of Avian Biology.

[ref-4] Adelman JS, Moyers SC, Farine DR, Hawley DM (2015). Feeder use predicts both acquisition and transmission of a contagious pathogen in a North American songbird. Proceedings of the Royal Society B: Biological Sciences.

[ref-5] Anadón A, Martinez-Larrañaga MR, Diaz MJ, Bringas P, Fernandez MC, Martinez MA, Fernandez-Cruz ML (1996). Pharmacokinetics of amoxicillin in broiler chickens. Avian Pathology.

[ref-6] Bailey MT, Dowd SE, Parry NM, Galley JD, Schauer DB, Lyte M (2010). Stressor exposure disrupts commensal microbial populations in the intestines and leads to increased colonization by *Citrobacter rodentium*. Infection and Immunity.

[ref-7] Bates D, Mächler M, Bolker B, Walker S (2015). Fitting linear mixed-effects models using lme4. Journal of Statistical Software.

[ref-8] Bates KA, Shelton JMG, Mercier VL, Hopkins KP, Harrison XA, Petrovan SO, Fisher MC (2019). Captivity and infection by the fungal pathogen *Batrachochytrium salamandrivorans* perturb the amphibian skin microbiome. Frontiers in Microbiology.

[ref-9] Bébéar C, Pereyre S, Peuchant O (2011). Mycoplasma pneumoniae: susceptibility and resistance to antibiotics. Future Microbiology.

[ref-10] Becker MH, Richards-Zawacki CL, Gratwicke B, Belden LK (2014). The effect of captivity on the cutaneous bacterial community of the critically endangered Panamanian golden frog (*Atelopus zeteki*). Biological Conservation.

[ref-11] Becker MH, Walke JB, Murrill L, Woodhams DC, Reinert LK, Rollins-Smith LA, Burzynski EA, Umile TP, Minbiole KP, Belden LK (2015). Phylogenetic distribution of symbiotic bacteria from Panamanian amphibians that inhibit growth of the lethal fungal pathogen *Batrachochytrium dendrobatidis*. Molecular Ecology.

[ref-12] Benjamini Y, Hochberg Y (1995). Controlling the false discovery rate: a practical and powerful approach to multiple testing. Journal of the Royal Statistical Society.

[ref-13] Bestion E, Jacob S, Zinger L, Di Gesu L, Richard M, White J, Cote J (2017). Climate warming reduces gut microbiota diversity in a vertebrate ectotherm. Nature Ecology & Evolution.

[ref-14] Bolyen E, Rideout JR, Dillon MR, Bokulich NA, Abnet CC, Al-Ghalith GA, Alexander H, Alm EJ, Arumugam M, Asnicar F, Bai Y, Bisanz JE, Bittinger K, Brejnrod A, Brislawn CJ, Brown CT, Callahan BJ, Caraballo-Rodríguez AM, Chase J, Cope EK, Silva RDa, Diener C, Dorrestein PC, Douglas GM, Durall DM, Duvallet C, Edwardson CF, Ernst M, Estaki M, Fouquier J, Gauglitz JM, Gibbons SM, Gibson DL, Gonzalez A, Gorlick K, Guo J, Hillmann B, Holmes S, Holste H, Huttenhower C, Huttley GA, Janssen S, Jarmusch AK, Jiang L, Kaehler BD, Kang KB, Keefe CR, Keim P, Kelley ST, Knights D, Koester I, Kosciolek T, Kreps J, Langille MGI, Lee J, Ley R, Liu Y-X, Loftfield E, Lozupone C, Maher M, Marotz C, Martin BD, McDonald D, McIver LJ, Melnik AV, Metcalf JL, Morgan SC, Morton JT, Naimey AT, Navas-Molina JA, Nothias LF, Orchanian SB, Pearson T, Peoples SL, Petras D, Preuss ML, Pruesse E, Rasmussen LB, Rivers A, Robeson MS, Rosenthal P, Segata N, Shaffer M, Shiffer A, Sinha R, Song SJ, Spear JR, Swafford AD, Thompson LR, Torres PJ, Trinh P, Tripathi A, Turnbaugh PJ, Ul-Hasan S, Hooft JJJvander, Vargas F, Vázquez-Baeza Y, Vogtmann E, Hippel Mvon, Walters W, Wan Y, Wang M, Warren J, Weber KC, Williamson CHD, Willis AD, Xu ZZ, Zaneveld JR, Zhang Y, Zhu Q, Knight R, Caporaso JG (2019). Reproducible, interactive, scalable and extensible microbiome data science using QIIME 2. Nature Biotechnology.

[ref-15] Boon E, Meehan CJ, Whidden C, Wong DH-J, Langille MG, Beiko RG (2014). Interactions in the microbiome: communities of organisms and communities of genes. FEMS Microbiology Reviews.

[ref-16] Bornbusch SL, Harris RL, Grebe NM, Roche K, Dimac-Stohl K, Drea CM (2021). Antibiotics and fecal transfaunation differentially affect microbiota recovery, associations, and antibiotic resistance in lemur guts. Animal Microbiome.

[ref-17] Broom LJ, Kogut MH (2018). The role of the gut microbiome in shaping the immune system of chickens. Veterinary Immunology and Immunopathology.

[ref-18] Callahan BJ, McMurdie PJ, Rosen MJ, Han AW, Johnson AJA, Holmes SP (2016). DADA2: High-resolution sample inference from Illumina amplicon data. Nature Methods.

[ref-19] Caporaso JG, Lauber CL, Walters WA, Berg-Lyons D, Huntley J, Fierer N, Owens SM, Betley J, Fraser L, Bauer M, Gormley N, Gilbert JA, Smith G, Knight R (2012). Ultra-high-throughput microbial community analysis on the Illumina HiSeq and MiSeq platforms. The ISME Journal.

[ref-20] Ceruelos AH, Romero-Quezada LC, Ledezma JR, Contreras LL (2019). Therapeutic uses of metronidazole and its side effects: an update. European Review for Medical and Pharmacological Sciences.

[ref-21] Cheng Y, Fox S, Pemberton D, Hogg C, Papenfuss AT, Belov K (2015). The Tasmanian devil microbiome—implications for conservation and management. Microbiome.

[ref-22] Clayton JB, Vangay P, Huang HU, Ward T, Hillmann BM, Al-Ghalith GA, Travis DA, Long HT, Van Tuan B, Van Minh V (2016). Captivity humanizes the primate microbiome. Proceedings of the National Academy of Sciences of the United States of America.

[ref-23] Connelly S, Subramanian P, Hasan NA, Colwell RR, Kaleko M (2018). Distinct consequences of amoxicillin and ertapenem exposure in the porcine gut microbiome. Anaerobe.

[ref-24] Cybulski W, Larsson P, Tjälve H, Kowalska-Pylka H, Sylla M, Semeniuk S (1996). Disposition of metronidazole in hens (*Gallus gallus*) and quails (*Coturnix coturnix japonica*): pharmacokinetics and whole-body autoradiography. Journal of Veterinary Pharmacology and Therapeutics.

[ref-25] Daskin JH, Alford RA (2012). Context-dependent symbioses and their potential roles in wildlife diseases. Proceedings of the Royal Society B: Biological Sciences.

[ref-26] Davidson GL, Somers SE, Wiley N, Johnson CN, Reichert MS, Ross RP, Stanton C, Quinn JL (2021). A time-lagged association between the gut microbiome, nestling weight and nestling survival in wild great tits. Journal of Animal Ecology.

[ref-27] Dorrestein GM, Tully TN, Dorrestein GM, Jones AK (2009). Passerines. Handbook of avian medicine, Second Edition.

[ref-28] Escallón C, Becker MH, Walke JB, Jensen RV, Cormier G, Belden LK, Moore IT (2017). Testosterone levels are positively correlated with cloacal bacterial diversity and the relative abundance of Chlamydiae in breeding male rufous-collared sparrows. Functional Ecology.

[ref-29] Faustino CR, Jennelle CS, Connolly V, Davis AK, Swarthout EC, Dhondt AA, Cooch EG (2004). Mycoplasma gallisepticum infection dynamics in a house finch population: seasonal variation in survival, encounter and transmission rate. Journal of Animal Ecology.

[ref-30] Fernandes AD, Macklaim JM, Linn TG, Reid G, Gloor GB (2013). ANOVA-like differential expression (ALDEx) analysis for mixed population RNA-Seq. PLOS ONE.

[ref-31] Fernandes AD, Reid JN, Macklaim JM, McMurrough TA, Edgell DR, Gloor GB (2014). Unifying the analysis of high-throughput sequencing datasets: characterizing RNA-seq, 16S rRNA gene sequencing and selective growth experiments by compositional data analysis. Microbiome.

[ref-32] Fox J, Weisberg S (2019). An R companion to applied regression.

[ref-33] Grodio JL, Dhondt KV, O’Connell PH, Schat KA (2008). Detection and quantification of *Mycoplasma gallisepticum* genome load in conjunctival samples of experimentally infected house finches (*Carpodacus mexicanus*) using real-time polymerase chain reaction. Avian Pathology.

[ref-34] Grond K, Sandercock BK, Jumpponen A, Zeglin LH (2018). The avian gut microbiota: community, physiology and function in wild birds. Journal of Avian Biology.

[ref-35] Harris EV, Roode JC de, Gerardo NM (2019). Diet–microbiome–disease: investigating diet’s influence on infectious disease resistance through alteration of the gut microbiome. PLOS Pathogens.

[ref-36] Hawley DM, Grodio J, Jr SF, Kirkpatrick L, Ley DH (2011). Experimental infection of domestic canaries (*Serinus canaria domestica*) with *Mycoplasma gallisepticum*: a new model system for a wildlife disease. Avian Pathology.

[ref-37] Hernandez J, Escallón C, Medina D, Vernasco BJ, Walke JB, Belden LK, Moore IT (2020). Cloacal bacterial communities of tree swallows (*Tachycineta bicolor*): similarity within a population, but not between pair-bonded social partners. PLOS ONE.

[ref-38] Hochachka WM, Dhondt AA (2000). Density-dependent decline of host abundance resulting from a new infectious disease. Proceedings of the National Academy of Sciences of the United States of America.

[ref-39] Honda K, Littman DR (2012). The microbiome in infectious disease and inflammation. Annual Review of Immunology.

[ref-40] Hooper LV, Littman DR, Macpherson AJ (2012). Interactions between the microbiota and the immune system. Science.

[ref-41] Ichinohe T, Pang IK, Kumamoto Y, Peaper DR, Ho JH, Murray TS, Iwasaki A (2011). Microbiota regulates immune defense against respiratory tract influenza A virus infection. Proceedings of the National Academy of Sciences of the United States of America.

[ref-42] Kabat AM, Srinivasan N, Maloy KJ (2014). Modulation of immune development and function by intestinal microbiota. Trends in Immunology.

[ref-43] Kalyana Chakravarthy S, Jayasudha R, Prashanthi GSai, Ali MH, Sharma S, Tyagi M, Shivaji S (2018). Dysbiosis in the gut bacterial microbiome of patients with uveitis, an inflammatory disease of the eye. Indian Journal of Microbiology.

[ref-44] Kelly LW, Williams GJ, Barott KL, Carlson CA, Dinsdale EA, Edwards RA, Haas AF, Haynes M, Lim YW, McDole T, Nelson CE, Sala E, Sandin SA, Smith JE, Vermeij MJA, Youle M, Rohwer F (2014). Local genomic adaptation of coral reef-associated microbiomes to gradients of natural variability and anthropogenic stressors. Proceedings of the National Academy of Sciences of the United States of America.

[ref-45] Keohane DM, Woods T, O’Connor P, Underwood S, Cronin O, Whiston R, O’Sullivan O, Cotter P, Shanahan F, Molloy MGM (2019). Four men in a boat: ultra-endurance exercise alters the gut microbiome. Journal of Science and Medicine in Sport.

[ref-46] Khosravi A, Mazmanian SK (2013). Disruption of the gut microbiome as a risk factor for microbial infections. Current Opinion in Microbiology.

[ref-47] Klomp JE, Murphy MT, Smith SB, McKay JE, Ferrera I, Reysenbach A-L (2008). Cloacal microbial communities of female spotted towhees *Pipilo maculatus*: microgeographic variation and individual sources of variability. Journal of Avian Biology.

[ref-48] Kogut MH, Lee A, Santin E (2020). Microbiome and pathogen interaction with the immune system. Poultry Science.

[ref-49] Kohl K (2012). Diversity and function of the avian gut microbiota. Journal of Comparative Physiology B: Biochemical, Systemic, and Environmental Physiology.

[ref-50] Kohl KD, Brun A, Bordenstein SR, Caviedes-Vidal E, Karasov WH (2018). Gut microbes limit growth in house sparrow nestlings (*Passer domesticus*) but not through limitations in digestive capacity. Integrative Zoology.

[ref-51] Kohl KD, Brun A, Magallanes M, Brinkerhoff J, Laspiur A, Acosta JC, Caviedes-Vidal E, Bordenstein SR (2017). Gut microbial ecology of lizards: insights into diversity in the wild, effects of captivity, variation across gut regions and transmission. Molecular Ecology.

[ref-52] Koskella B, Bergelson J (2020). The study of host–microbiome (co)evolution across levels of selection. Philosophical Transactions of the Royal Society B.

[ref-53] Kraemer SA, Ramachandran A, Perron GG (2019). Antibiotic pollution in the environment: from microbial ecology to public policy. Microorganisms.

[ref-54] Lavrinienko A, Hämäläinen A, Hindström R, Tukalenko E, Boratyński Z, Kivisaari K, Mousseau TA, Watts PC, Mappes T (2021). Comparable response of wild rodent gut microbiome to anthropogenic habitat contamination. Molecular Ecology.

[ref-55] Lazar V, Ditu L-M, Pircalabioru GG, Gheorghe I, Curutiu C, Holban AM, Picu A, Petcu L, Chifiriuc MC (2018). Aspects of gut microbiota and immune system interactions in infectious diseases, immunopathology, and cancer. Frontiers in Immunology.

[ref-56] Leon AE, Hawley DM (2017). Host responses to pathogen priming in a natural songbird host. Ecohealth.

[ref-57] Ley DH, Hawley DM, Geary SJ, Dhondt AA (2016). House finch (*Haemorhous mexicanus*) conjunctivitis, and *Mycoplasma* spp. isolated from North American wild birds, 1994–2015. Journal of Wildlife Diseases.

[ref-58] Maurice CF, Knowles SCL, Ladau J, Pollard KS, Fenton A, Pedersen AB, Turnbaugh PJ (2015). Marked seasonal variation in the wild mouse gut microbiota. The ISME Journal.

[ref-59] McKnight DT, Huerlimann R, Bower DS, Schwarzkopf L, Alford RA, Zenger KR (2019). Methods for normalizing microbiome data: an ecological perspective. Methods in Ecology and Evolution.

[ref-60] Murray MH, Lankau EW, Kidd AD, Welch CN, Ellison T, Adams HC, Lipp EK, Hernandez SM (2020). Gut microbiome shifts with urbanization and potentially facilitates a zoonotic pathogen in a wading bird. PLOS ONE.

[ref-61] Nakamura YK, Metea C, Karstens L, Asquith M, Gruner H, Moscibrocki C, Lee I, Brislawn CJ, Jansson JK, Rosenbaum JT, Lin P (2016). Gut microbial alterations associated with protection from autoimmune uveitis. Investigative Ophthalmology & Visual Science.

[ref-62] Oksanen J, Simpson GL, Blanchet FG, Friendly M, Kindt R, Legendre P, Minchin PR, O’Hara RB, Solymos P, Stevens MHH, Szoecs E, Wagner H, Barbour M, Bedward M, Bolker B, Borcard D, Carvalho G, CHirico M, De Caceres M, Durand S, Antoniazi Evangelista HB, FitzJohn R, Friendly M, Furneaux B, Hannigan G, Hill MO, Lahti L, McGlinn D, Ouelette M-H, Ribeiro Cunha E, Smith T, Stier A, Ter Braak CJF, Weedon J (2020). https://CRAN.R-project.org/package=vegan.

[ref-63] Oliveira BCM, Murray M, Tseng F, Widmer G (2020). The fecal microbiota of wild and captive raptors. Animal Microbiome.

[ref-64] Oliver KM, Smith AH, Russell JA (2014). Defensive symbiosis in the real world–advancing ecological studies of heritable, protective bacteria in aphids and beyond. Functional Ecology.

[ref-65] Pallav K, Dowd SE, Villafuerte J, Yang X, Kabbani T, Hansen J, Dennis M, Leffler DA, Newburg DS, Kelly CP (2014). Effects of polysaccharopeptide from *Trametes versicolor* and amoxicillin on the gut microbiome of healthy volunteers: a randomized clinical trial. Gut Microbes.

[ref-66] Paradis E, Schliep K (2019). ape 5.0: an environment for modern phylogenetics and evolutionary analysis in R. Bioinformatics.

[ref-67] Pélissier M-A, Vasquez N, Balamurugan R, Pereira E, Dossou-Yovo F, Suau A, Pochart P, Magne F (2010). Metronidazole effects on microbiota and mucus layer thickness in the rat gut. FEMS Microbiology Ecology.

[ref-68] R Development Core Team (2015). http://www.gbif.org/resource/81287.

[ref-69] Rosshart SP, Vassallo BG, Angeletti D, Hutchinson DS, Morgan AP, Takeda K, Hickman HD, McCulloch JA, Badger JH, Ajami NJ (2017). Wild mouse gut microbiota promotes host fitness and improves disease resistance. Cell.

[ref-70] R Studio Team (2020).

[ref-71] Salgado-Flores A, Tveit AT, Wright A-D, Pope PB, Sundset MA (2019). Characterization of the cecum microbiome from wild and captive rock ptarmigans indigenous to Arctic Norway. PLOS ONE.

[ref-72] San Juan PA, Castro I, Dhami MK (2021). Captivity reduces diversity and shifts composition of the Brown Kiwi microbiome. Animal Microbiome.

[ref-73] Sarkar A, Harty S, Johnson KV-A, Moeller AH, Archie EA, Schell LD, Carmody RN, Clutton-Brock TH, Dunbar RI, Burnet PW (2020). Microbial transmission in animal social networks and the social microbiome. Nature Ecology & Evolution.

[ref-74] Schokker D, Jansman AJ, Veninga G, de Bruin N, Vastenhouw SA, de Bree FM, Bossers A, Rebel JM, Smits MA (2017). Perturbation of microbiota in one-day old broiler chickens with antibiotic for 24 h negatively affects intestinal immune development. BMC Genomics.

[ref-75] Schuijt TJ, Lankelma JM, Scicluna BP, eMelo F de S, Roelofs JJ, de Boer JD, Hoogendijk AJ, de Beer R, de Vos A, Belzer C (2016). The gut microbiota plays a protective role in the host defence against pneumococcal pneumonia. Gut.

[ref-76] Shukla SD, Budden KF, Neal R, Hansbro PM (2017). Microbiome effects on immunity, health and disease in the lung. Clinical & Translational Immunology.

[ref-77] Song SJ, Sanders JG, Delsuc F, Metcalf J, Amato K, Taylor MW, Mazel F, Lutz HL, Winker K, Graves GR, Humphrey G, Gilbert JA, Hackett SJ, White KP, Skeen HR, Kurtis SM, Withrow J, Braile T, Miller M, McCracken KG, Maley JM, Ezenwa VO, Williams A, Blanton JM, McKenzie VJ, Knight R (2020). Comparative analyses of vertebrate gut microbiomes reveal convergence between birds and bats. mBio.

[ref-78] Sydenstricker KV, Dhondt AA, Ley DH, Kollias GV (2005). Re-exposure of captive house finches that recovered from *Mycoplasma gallisepticum* infection. Journal of Wildlife Diseases.

[ref-79] Teyssier A, Rouffaer LO, Hudin NS, Strubbe D, Matthysen E, Lens L, White J (2018). Inside the guts of the city: urban-induced alterations of the gut microbiota in a wild passerine. Science of the Total Environment.

[ref-80] Thomason CA, Leon A, Kirkpatrick LT, Belden LK, Hawley DM (2017). Eye of the Finch: characterization of the ocular microbiome of house finches in relation to mycoplasmal conjunctivitis. Environmental Microbiology.

[ref-81] Trevelline BK, Fontaine SS, Hartup BK, Kohl KD (2019). Conservation biology needs a microbial renaissance: a call for the consideration of host-associated microbiota in wildlife management practices. Proceedings of the Royal Society B: Biological Sciences.

[ref-82] Videvall E, Strandh M, Engelbrecht A, Cloete S, Cornwallis CK (2018). Measuring the gut microbiome in birds: comparison of faecal and cloacal sampling. Molecular Ecology Resources.

[ref-83] Vieira WA, Pretorius E (2010). The impact of asthma on the gastrointestinal tract (GIT). Journal of Asthma and Allergy.

[ref-84] Vinkler M, Leon AE, Kirkpatrick L, Dalloul RA, Hawley DM (2018). Differing house finch cytokine expression responses to original and evolved isolates of *Mycoplasma gallisepticum*. Frontiers in Immunology.

[ref-85] Vlčková K, Shutt-Phillips K, Heistermann M, Pafčo B, Petrželková KJ, Todd A, Modrý D, Nelson KE, Wilson BA, Stumpf RM, White BA, Leigh SR, Gomez A (2018). Impact of stress on the gut microbiome of free-ranging western lowland gorillas. Microbiology.

[ref-86] Walke JB, Belden LK (2016). Harnessing the microbiome to prevent fungal infections: lessons from amphibians. PLOS Pathogens.

[ref-87] Wang J, Li F, Wei H, Lian Z-X, Sun R, Tian Z (2014). Respiratory influenza virus infection induces intestinal immune injury via microbiota-mediated Th17 cell–dependent inflammation. Journal of Experimental Medicine.

[ref-88] Weitzman CL, Rostama B, Thomason CA, May M, Belden LK, Hawley DM (2021). Experimental test of microbiome protection across pathogen doses reveals importance of resident microbiome composition. FEMS Microbiology Ecology.

[ref-89] Weyrich LS, Feaga HA, Park J, Muse SJ, Safi CY, Rolin OY, Young SE, Harvill ET (2014). Resident microbiota affect *Bordetella pertussis* infectious dose and host specificity. The Journal of Infectious Diseases.

[ref-90] Wilks J, Golovkina T (2012). Influence of microbiota on viral infections. PLOS Pathogens.

[ref-91] Wise MG, Siragusa GR (2007). Quantitative analysis of the intestinal bacterial community in one-to three-week-old commercially reared broiler chickens fed conventional or antibiotic-free vegetable-based diets. Journal of Applied Microbiology.

[ref-92] Worsley SF, Davies CS, Mannarelli M-E, Hutchings MI, Komdeur J, Burke T, Dugdale HL, Richardson DS (2021). Gut microbiome composition, not alpha diversity, is associated with survival in a natural vertebrate population. Animal Microbiome.

[ref-93] Xiong W, Wang Y, Sun Y, Ma L, Zeng Q, Jiang X, Li A, Zeng Z, Zhang T (2018). Antibiotic-mediated changes in the fecal microbiome of broiler chickens define the incidence of antibiotic resistance genes. Microbiome.

[ref-94] Yuan ML, Dean SH, Longo AV, Rothermel BB, Tuberville TD, Zamudio KR (2015). Kinship, inbreeding and fine-scale spatial structure influence gut microbiota in a hindgut-fermenting tortoise. Molecular Ecology.

