# Supplementary material for: Antibiotic perturbation of gut bacteria does not significantly alter host responses to ocular disease in a songbird species
Source: PeerJ. 2022 Jun 10;10:e13559. doi: 10.7717/peerj.13559 (PMC9190666; doi:10.7717/peerj.13559)
Supplement: Supplemental Information 1 [file peerj-10-13559-s001.docx]

**Supplemental Material (Part I)**

**TITLE:**

Antibiotic perturbation of gut bacteria does not significantly alter host responses to ocular disease in a songbird species

**AUTHORS:**

Chava L. Weitzman*, Lisa K. Belden, Meghan May, Marissa M. Langager, Rami A. Dalloul, and Dana M. Hawley

* weitzman.chava@gmail.com

**Summary:**

Additional methods and results are provided for serology testing of birds in the experiment described in the *Main Text*. Detailed sample sizes are provided for DNA extraction of ocular swab samples (used for MG load analyses, Table S1). We provide extended results on comparisons of cloacal bacterial communities from wild and captive house finches sampled during the experiment. Contains Tables S1–4 and Figures S1–5.

**Methods**

*Serology Testing*

On day 20 of the experiment described in the *Main Text*, we collected blood samples via brachial venipuncture into heparinized capillary tubes. Plasma was separated off and stored at –20 ˚C. We tested samples representing all four treatment groups (n = 44) for the presence of anti-MG antibodies via enzyme-linked immunosorbent assay (ELISA) as per Hawley et al. (2011). After verifying that no MG-control birds seroconverted (conservative raw OD cut-off of 0.0671, Weitzman et al., 2021), we analyzed ELISA data (optical density) from birds inoculated with MG as a continuous variable in linear models to detect if the gut microbiome antibiotics treatment affected birds’ antibody response.

**Table S1. Ocular swab extraction sample sizes used for MGC2 qPCR.** Sample sizes given for each treatment on each post-inoculation day, separated by sex (F = female, M = male).

| **Treatment 🡪** | **Antibiotics – MG –** | | **Antibiotics + MG –** | | **Antibiotics + MG +** | | **Antibiotics – MG +** | |
| --- | --- | --- | --- | --- | --- | --- | --- | --- |
| **Post-inoculation Day** | **F** | **M** | **F** | **M** | **F** | **M** | **F** | **M** |
| **-1** |  |  |  |  | 4 | 4 | 4 | 4 |
| **3** | 4 | 4 | 4 | 4 | 5 | 5 | 5 | 5 |
| **7** |  |  |  |  | 5 | 5 | 5 | 5 |
| **13** |  |  |  |  | 5 | 5 | 5 | 5 |

**Results**

*Serology Testing*

We analyzed ELISA results from MG-inoculated birds to determine if perturbing the gut microbiome affected birds’ adaptive immune response, specifically antibody production in the plasma. Nearly all MG-inoculated birds had detectable antibodies on day 20 (averages ± SD,

non-treated: 0.0943 ± 0.0209; antibiotics: 0.0913 ± 0.0212). The two MG-inoculated groups did not differ in antibody levels on day 20 (*F*_1,28_ = 0.159, *p* = 0.7).

*Amplicon Sequencing*

Amplicon sequencing of our 65 house finch cloacal swab samples resulted in 5,877,887 total reads. After error correction (filterandtrim), removal of spurious reads (dada), and removal of chimeric reads, 4,948,085 reads remained (84% of the total), 60.6% of which were identified as either non-bacterial, chloroplast, or mitochondrial. Thus, 1,951,109 bacterial reads were available in our samples for analyses of community differences.

**Table S2.** Results of beta diversity analyses comparing cloacal bacterial communities between birds given oral antibiotics and those in the antibiotics-control treatment. Paired data are from birds sampled on day –1 and day 7. **Bold** denotes significant differences between the groups.

| **Response** | **Test** | **Sum of Squares** | ***F*** | ***R^2^*** | ***P*** |
| --- | --- | --- | --- | --- | --- |
| **Bray–Curtis** |  |  |  |  |  |
|  | PERMANOVA | 0.85 | 2.39 | 5.0% | > 0.5 |
|  | Beta Dispersion | **0.016** | **4.48** |  | **0.04** |
|  |  |  |  |  |  |
| **Bray–Curtis, paired** | |  |  |  |  |
|  | ANOVA | 0.046 | 1.48 |  | 0.2 |
|  |  |  |  |  |  |
| **Jaccard** |  |  |  |  |  |
|  | PERMANOVA | 0.79 | 1.93 | 4.1% | > 0.5 |
|  | Beta Dispersion | 0.006 | 3.82 |  | 0.06 |
|  |  |  |  |  |  |
| **Jaccard, paired** | |  |  |  |  |
|  | ANOVA | 0.02 | 1.12 |  | 0.3 |
|  |  |  |  |  |  |

**Table S3.** Results of comparisons between cloacal bacterial communities collected from wild birds in the field and from house finches in the microbiome-control group in our experiment on day –1. ASV richness and Faith’s phylogenetic diversity values were from communities rarefied to 5000 reads per sample. **Bold** denotes significant differences between the groups.

| **Response** | **Test** | **Sum of Squares** | ***F*** | ***R^2^*** | ***P*** |
| --- | --- | --- | --- | --- | --- |
| **ASV Richness** | ANOVA | **4.14** | **6.93** |  | **0.01** |
|  |  |  |  |  |  |
| **Phylogenetic Diversity** | ANOVA | 0.69 | 1.85 |  | 0.18 |
|  |  |  |  |  |  |
| **Weighted UniFrac** |  |  |  |  |  |
|  | PERMANOVA | **0.59** | **3.64** | **9.9%** | **0.005** |
|  | Beta Dispersion | 0.019 | 1.77 |  | 0.18 |
|  |  |  |  |  |  |
| **Unweighted UniFrac** |  |  |  |  |  |
|  | PERMANOVA | **0.95** | **3.98** | **10.8%** | **0.001** |
|  | Beta Dispersion | **0.013** | **5.07** |  | **0.04** |
|  |  |  |  |  |  |
| **Bray–Curtis** |  |  |  |  |  |
|  | PERMANOVA | **1.47** | **3.92** | **10.6%** | **0.001** |
|  | Beta Dispersion | 0.0003 | 0.15 |  | 0.68 |
|  |  |  |  |  |  |
| **Jaccard** |  |  |  |  |  |
|  | PERMANOVA | **1.08** | **2.56** | **7.2%** | **0.001** |
|  | Beta Dispersion | 0.0002 | 0.25 |  | 0.64 |
|  |  |  |  |  |  |

**Figure S1. Abundant bacterial phyla and genera in wild and captive** **house finch cloacal swab samples.** Phyla included represent, on average, at least 1% of the reads per group of samples. Genera included represent, on average, at least 2% of the reads per group of samples.


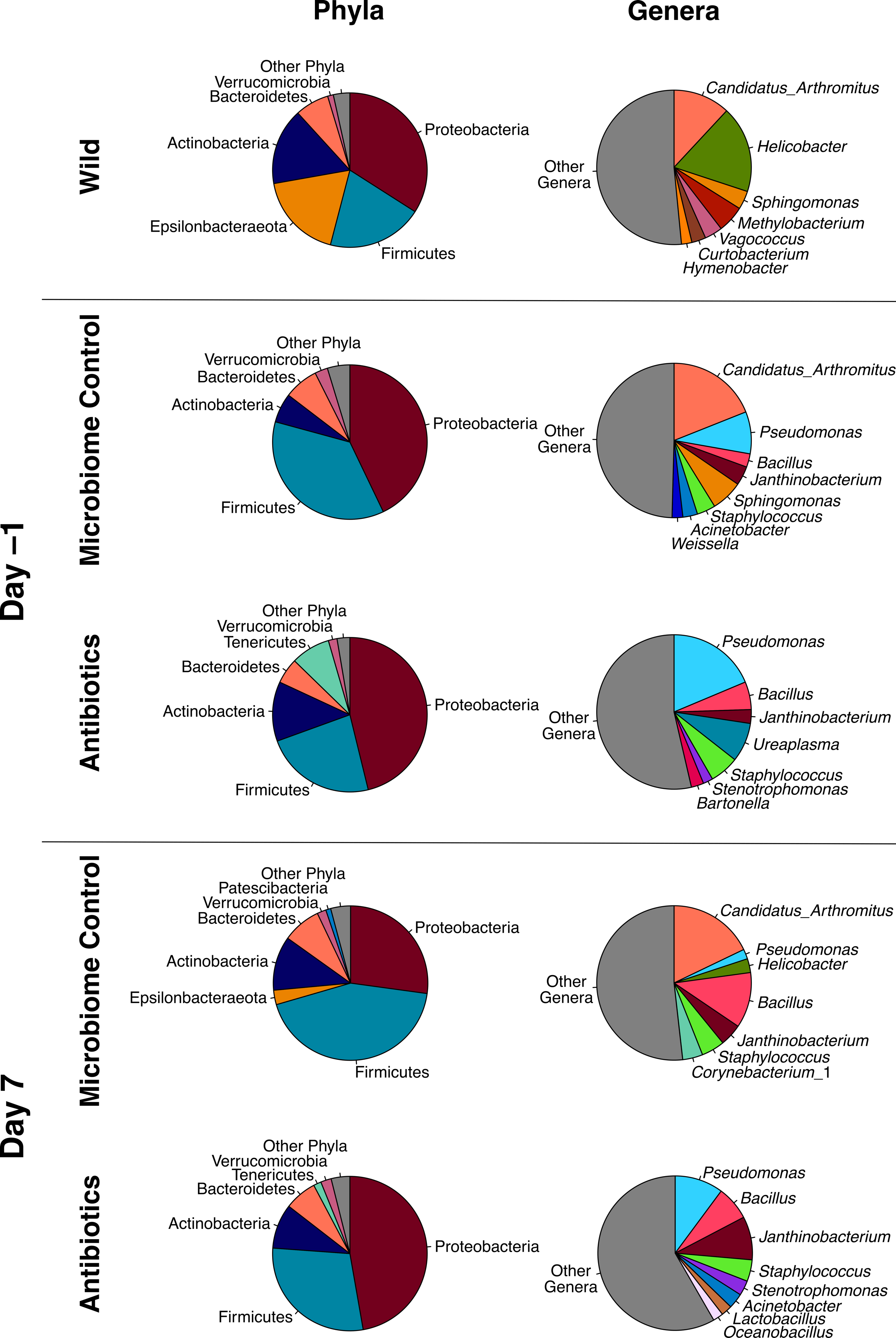


**Figure S2. Beta diversity in house finch cloacal bacterial communities in captive birds.** Principal coordinates analysis on unweighted and weighted UniFrac distances, Jaccard distance, and Bray–Curtis dissimilarity. Triangles indicate birds given oral antibiotics. Circles indicate birds not given oral antibiotics. Colors indicate sampling day and antibiotics treatment.


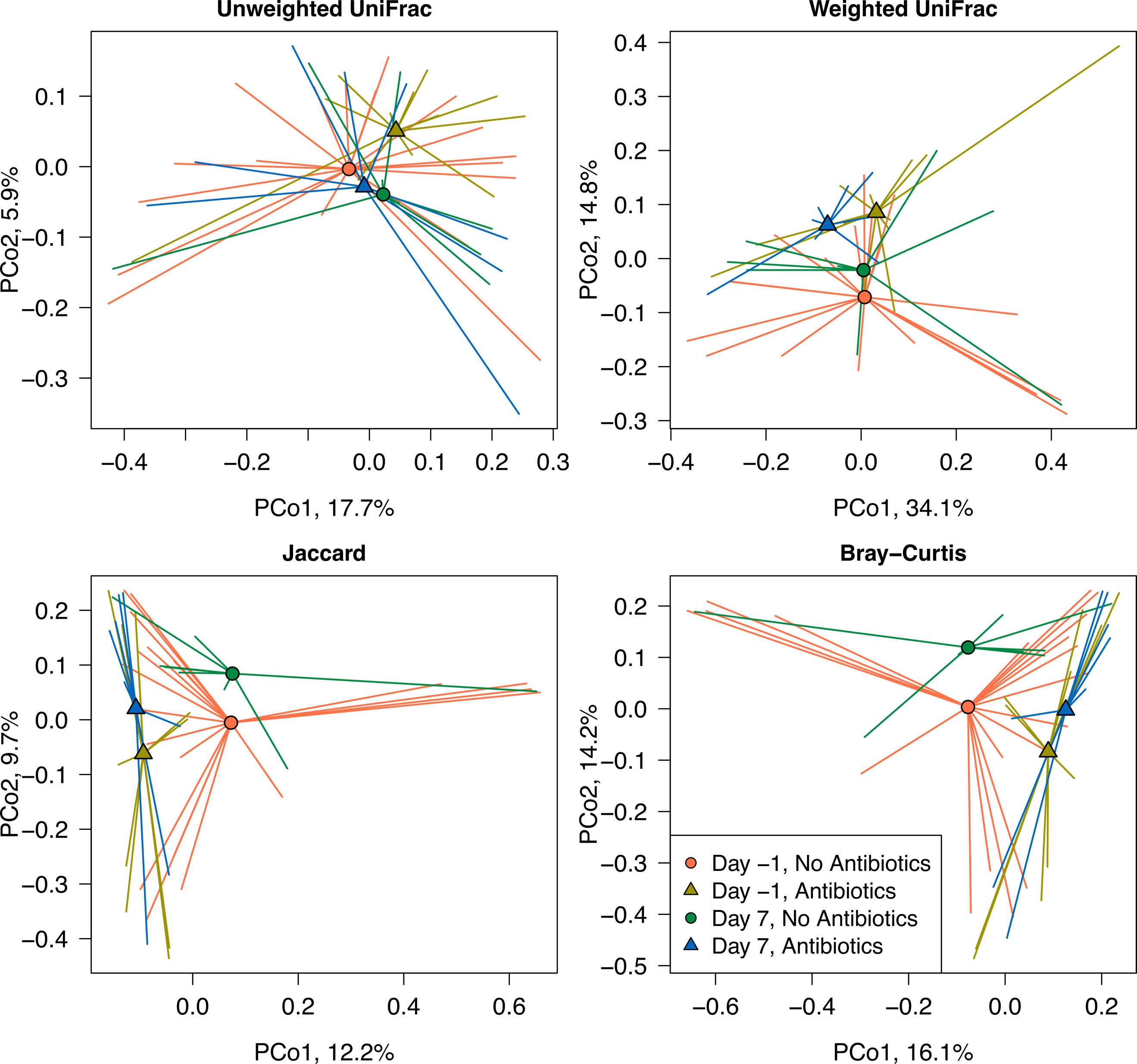


**Figure S3.** **ASV richness (A) and phylogenetic diversity (B) in wild and captive house finch cloacal swab samples.** Asterisk signifies significant difference between groups.


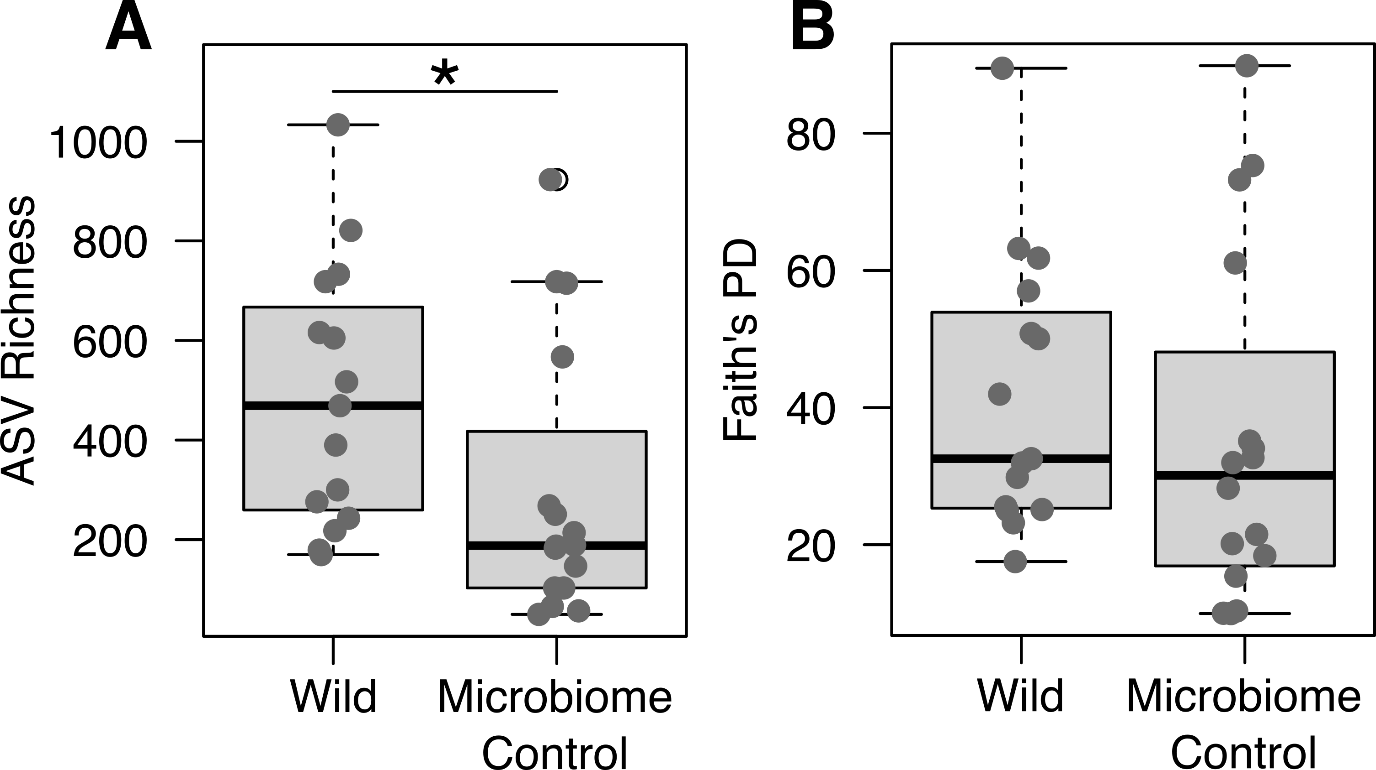


**Figure S4. Beta diversity in house finch cloacal bacterial communities from wild and captive samples,** comparing undisturbed communities in captive house finches (day –1, no antibiotics) with samples from wild birds. Principal coordinates analysis on weighted and unweighted UniFrac distances, Jaccard distance, and Bray–Curtis dissimilarity. Teal circles indicate captive birds. Orange triangles indicate wild birds.


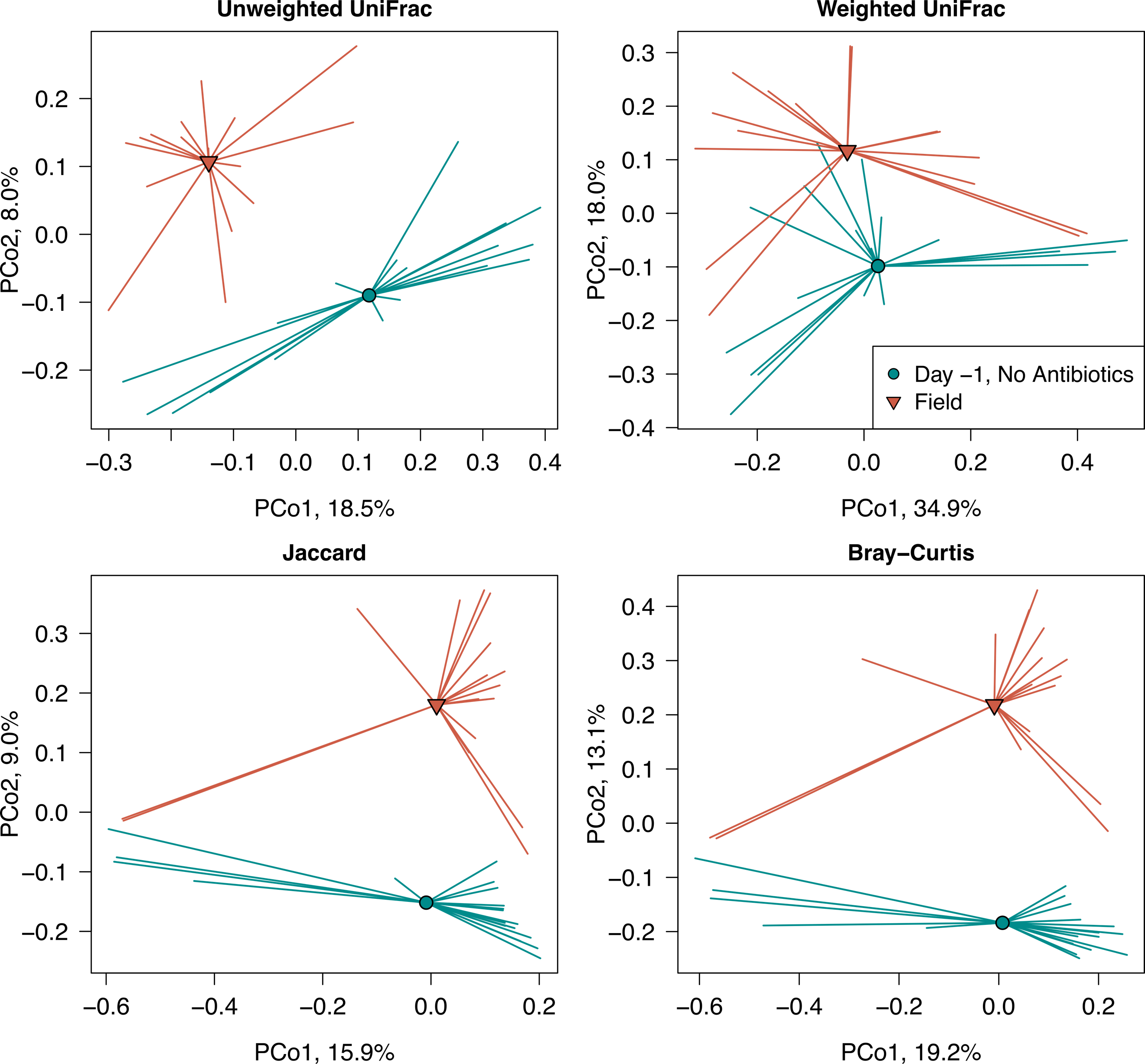


**Figure S5.** Venn diagram of core genera (85% prevalence cut-off) in wild and captive house finch cloacal swab samples.


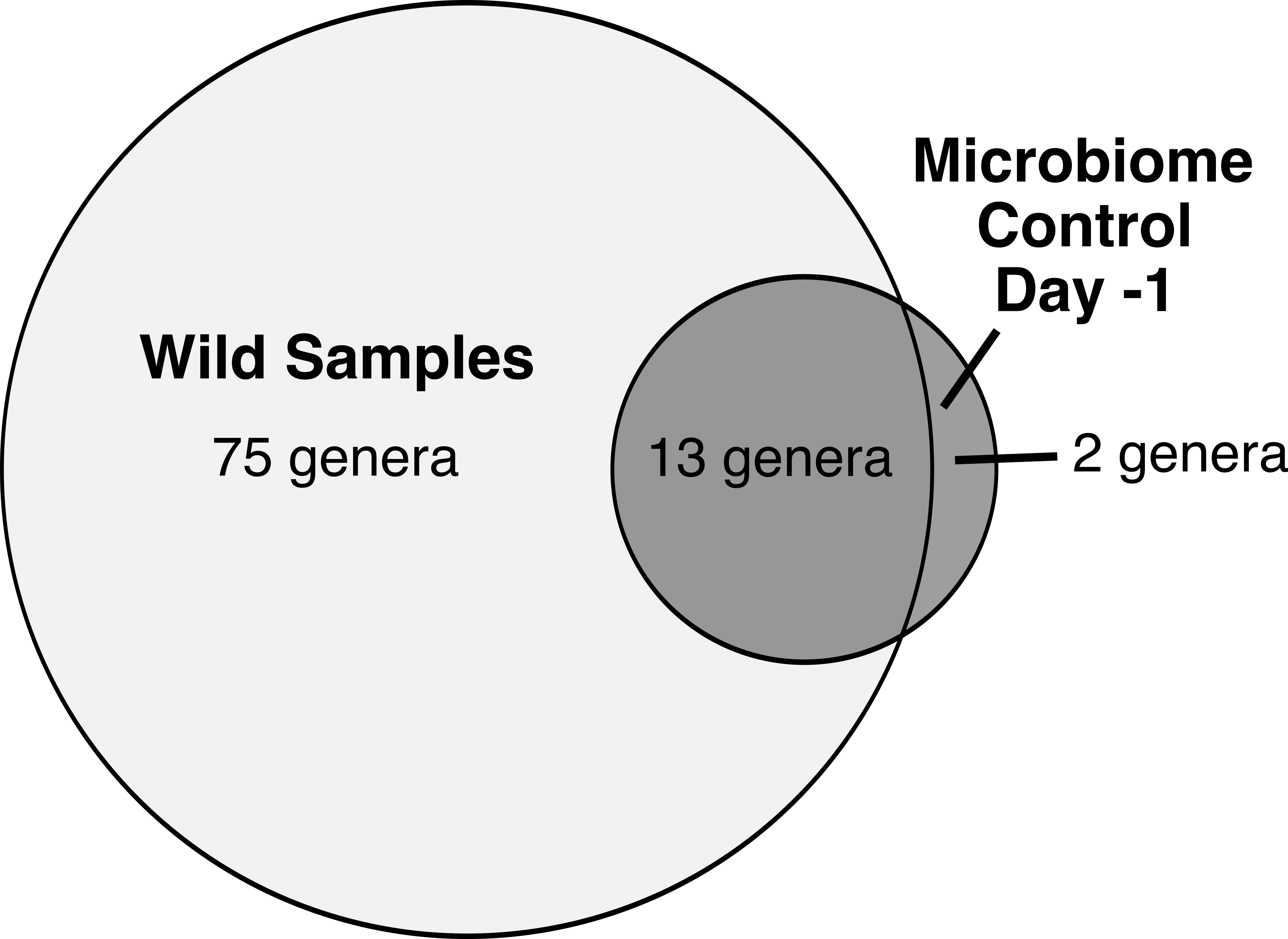


**References**

Hawley DM, Grodio J, Jr SF, Kirkpatrick L, Ley DH. 2011. Experimental infection of domestic canaries (*Serinus canaria domestica*) with *Mycoplasma gallisepticum*: a new model system for a wildlife disease. *Avian Pathology* 40:321–327. DOI: 10.1080/03079457.2011.571660.

Weitzman CL, Rostama B, Thomason CA, May M, Belden LK, Hawley DM. 2021. Experimental test of microbiome protection across pathogen doses reveals importance of resident microbiome composition. *FEMS Microbiology Ecology* 97:fiab141. DOI: 10.1093/femsec/fiab141.
