# Supplementary material for: Antibiotic perturbation of gut bacteria does not significantly alter host responses to ocular disease in a songbird species
Source: PeerJ. 2022 Jun 10;10:e13559. doi: 10.7717/peerj.13559 (PMC9190666; doi:10.7717/peerj.13559)
Supplement: Supplemental Information 2 [file peerj-10-13559-s002.docx]

**Supplemental Material (Part II)**

**Cloacal and intestinal swab communities**

**TITLE:**

Antibiotic perturbation of gut bacteria does not significantly alter host responses to ocular

disease in a songbird species

**AUTHORS:**

Chava L. Weitzman*, Lisa K. Belden, Meghan May, Marissa M. Langager, Rami A. Dalloul, and Dana M. Hawley

* weitzman.chava@gmail.com

**Summary:**

Contains methods and results of cloacal and intestinal swab sampling and sequencing from house finches conducted in 2014/2015. Contains Table S5 and Figure S6.

**Methods**

*Cloacal and Intestinal Swab Bacteria*

We use paired microbiome data from house finch cloacal and intestinal swab samples used in a prior study to provide preliminary insight into the extent to which cloacal swabs represent bacterial communities higher in the gastro-intestinal tract. In the winter of 2014, swab samples were collected from the cloaca and lower intestine of five house finches to compare bacterial communities between the two regions. These samples were collected at the culmination of a separate study (Leon and Hawley, 2017), 29 days after they were inoculated with a dose of *Mycoplasma gallisepticum*, and after all birds recovered from any mycoplasmal conjunctivitis experienced during the experiment. Cloacal swab samples were collected from five house finches using individually-wrapped, sterile swabs, after which, birds were euthanized with a lethal dose of carbon dioxide gas. Birds were dissected, and their lower intestines were swabbed. All swab samples were placed in 1.5 mL microcentrifuge tubes and frozen at –20 ˚C until DNA extraction.

DNA was extracted from swabs using the Qiagen DNeasy Blood and Tissue kit using the protocol for Gram-positive bacteria. Library prep for Illumina MiSeq sequencing was conducted as outlined in the *Main Text*. In total, we obtained 16S rRNA amplicons of four cloacal and four intestinal swab samples, including pairs of samples from three birds. Sequences are openly available on figshare (DOI: 10.6084/m9.figshare.16766602).

Single-end sequence reads were processed to ASVs in QIIME2 and R as outlined in the *Main Text.* Data were rarefied to the lowest sampling depth (18,972 reads per sample). We used QIIME2 to calculate core diversity metrics, including ASV richness, evenness, Shannon’s diversity, Faith’s phylogenetic diversity, and beta diversity metrics of Jaccard, Bray–Curtis, and weighted and unweighted UniFrac distances. We used Kruskal–Wallis tests to compare alpha diversity, and PERMANOVAs to compare beta diversity, between cloacal and intestinal swab bacterial communities.

**Results**

*Cloacal and Intestinal Swab Bacteria*

Based on our limited data from cloacal and intestinal swab samples from house finches, bacterial communities in the cloaca loosely represent those in the lower gastro-intestinal tract. The majority of all eight communities consisted of *Cellulomonas* and *Pseudomonas* (Fig. S6). Communities only significantly differed in Pielou’s evenness (Table S5). Additional, non-significant trends were detected in Shannon’s diversity and weighted UniFrac.

**Table S5.** Comparisons between cloacal and intestinal bacterial communities in house finches based on metrics of alpha and beta diversity. **Bold** indicates p-values below 0.1.

| Alpha Diversity | | | | | |
| --- | --- | --- | --- | --- | --- |
| **Dependent** | **Test** | **Cloacal**  **(avg ± SD)** | **Intestinal**  **(avg ± SD)** | **H** | **P** |
| Richness | Kruskal–Wallis | 80.5 ± 6.03 | 80 ± 3.92 | 0.0833 | 0.77 |
| Shannon | Kruskal–Wallis | 3.10 ± 0.171 | 3.52 ± 0.313 | **3** | **0.0833** |
| Evenness | Kruskal–Wallis | 0.490 ± 0.0235 | 0.557 ± 0.0442 | **4.083** | **0.043** |
| Faith PD | Kruskal–Wallis | 6.67 ± 0.716 | 6.74 ± 0.259 | 0 | 1 |
|  |  |  |  |  |  |
| Beta Diversity | | | | | |
| **Dependent** | **Test** |  |  | **pseudo-F** | **P** |
| Jaccard | PERMANOVA |  |  | 1.155 | 0.12 |
| Bray–Curtis | PERMANOVA |  |  | 1.26225 | 0.272 |
| Unweighted UniFrac | PERMANOVA |  |  | 1.0943 | 0.342 |
| Weighted UniFrac | PERMANOVA |  |  | **4.02995** | **0.099** |

**Figure S6.** Bacterial genera in four cloacal and four intestinal swabs from house finches. Each bar represents one sample. Only genera constituting >2% relative abundance within the sample were identified. Samples are labeled by bird ID and sample type (C = cloacal, I = intestinal). Three samples are paired (i.e., cloacal and intestinal swab data from the same individual), while one sample of each type is unpaired.

**
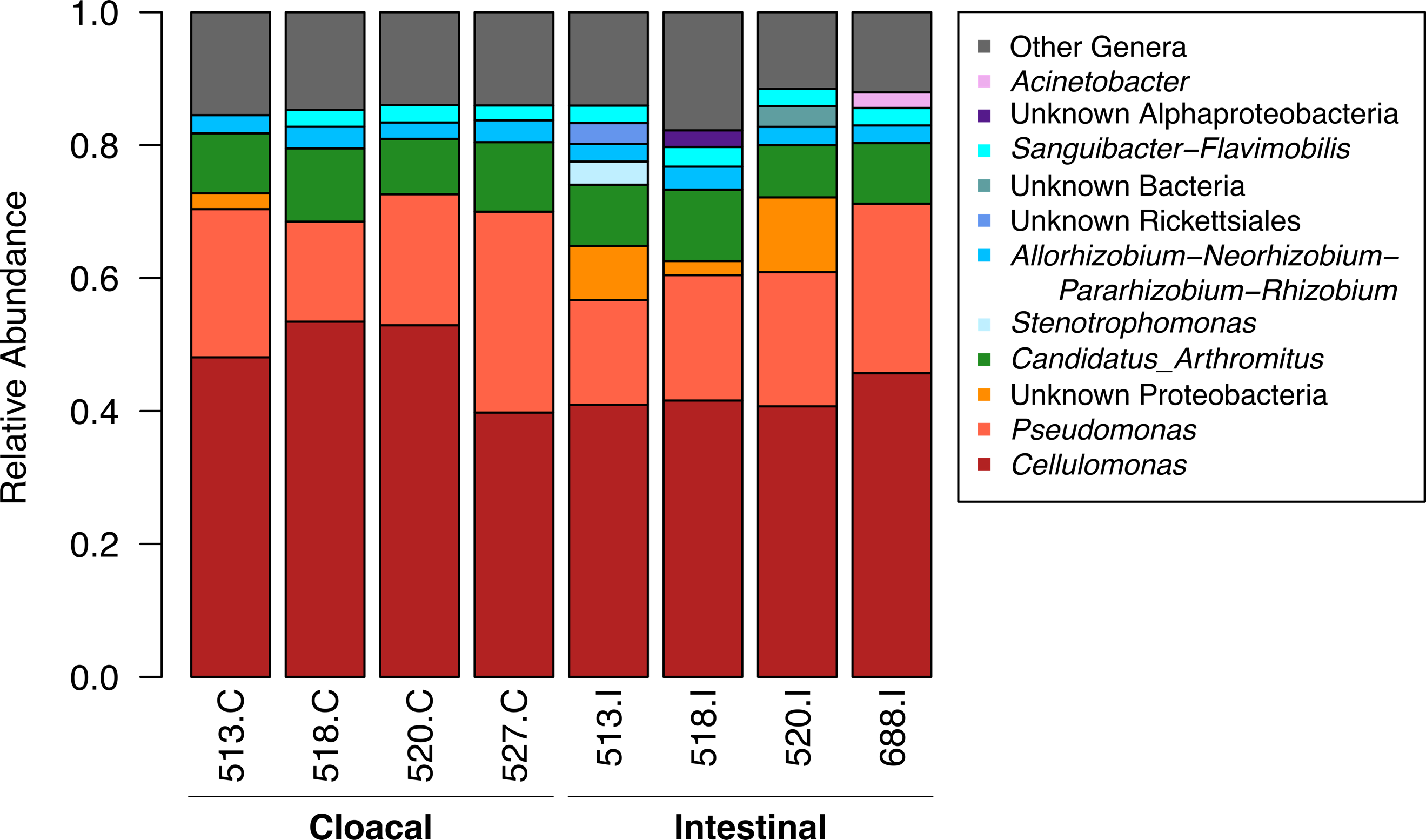
**

**References**

Leon AE, Hawley DM. 2017. Host responses to pathogen priming in a natural songbird host. *Ecohealth* 14:793–804. DOI: 10.1007/s10393-017-1261-x.
